# USP2 is an androgen-repressed survival factor that stabilises oncoproteins to facilitate therapy resistance in prostate cancer

**DOI:** 10.1038/s41419-026-08923-7

**Published:** 2026-06-02

**Authors:** Danielle Meiwen Fang, Raj K. Shrestha, Beatriz German, Razia Rahman, Scott L. Townley, Jianling Xie, Adrienne R. Hanson, Richard Iggo, Zeyad D. Nassar, Madison Helm, Shashikanth Marri, Alex D. Colella, Giles Best, Margaret M. Centenera, Wayne D. Tilley, Tim K. Chataway, Leigh Ellis, Lisa M. Butler, Luke A. Selth

**Affiliations:** 1 Flinders Health and Medical Research Institute, College of Medicine and Public Health, Flinders University, Adelaide, SA Australia; 2 Dame Roma Mitchell Cancer Research Laboratories, Adelaide Medical School, University of Adelaide, Adelaide, SA Australia; 3 Freemasons Centre for Male Health and Wellbeing, Flinders University, Bedford Park, SA Australia; 4 Center for Prostate Disease Research, Murtha Cancer Center Research Program, Department of Surgery, Uniformed Services University of the Health Sciences, Bethesda, MD USA; 5 The Henry M. Jackson Foundation for the Advancement of Military Medicine Inc., Bethesda, MD USA; 6 Genitourinary Malignancies Branch, Center for Cancer Research, National Cancer Institute, Bethesda, MD USA; 7 Guangdong Engineering Research Center of Low-Carbon Synthetic Biotechnology, School of Biology and Biological Engineering, South China University of Technology, University Town, Guangzhou, China; 8 Institut Bergonié, University of Bordeaux, INSERM, Bordeaux, France; 9 South Australian Health and Medical Research Institute, Adelaide, SA Australia; 10 South Australian immunoGENomics Cancer Institute (SAiGENCI), University of Adelaide, Adelaide, SA Australia; 11 Flinders Proteomics Facility, Flinders University, Bedford Park, SA Australia; 12 Adelaide Medical School, Faculty of Health and Medical Sciences, The University of Adelaide, Adelaide, SA Australia

**Keywords:** Prostate cancer, Oncogenes, Ubiquitylation

## Abstract

In prostate cancer, the development of resistance to androgen receptor (AR)-targeted therapies (ATTs) and chemotherapies is associated with the emergence of new phenotypic states with altered molecular features. Here, we identify ubiquitin-specific peptidase 2 (USP2) as a mediator of this phenomenon. USP2 is induced in response to ATTs and more highly expressed in aggressive, castration-resistant prostate tumours that have lost dependence on AR as an oncogenic driver. Overexpression of USP2 in prostate cancer cells elicited elevated rates of glycolysis, neuroendocrine features and resistance to docetaxel, an important chemotherapeutic agent for advanced prostate cancer. USP2 stabilised key oncoproteins, including Aurora kinase A, cyclin D1 and fatty acid synthase. An unbiased proteomic approach identified potential new USP2 substrates and a USP2-regulated proteome that is associated with metastatic disease. Genetic or pharmacological targeting of USP2 caused cell death in human and mouse models of aggressive prostate cancer and the USP2 inhibitor ML364 exhibited potent anti-tumour effects against an orthotopic xenograft model of AR-null disease. Collectively, this study identifies USP2 as a critical regulator of proteins involved in prostate cancer progression and therapy resistance, positioning it as a viable therapeutic target.

## Introduction

The growth of prostate cancer (PCa) is highly dependent on the androgen receptor (AR) signalling axis. Hence, metastatic PCa is treated with AR-targeted therapies (ATTs), which either block androgen production or act as AR antagonists [1]. While initially effective, patients inevitably develop resistance to ATTs and progress to castration-resistant prostate cancer (CRPC) [1]. The mechanisms underlying resistance to ATTs are diverse and complex [2, 3]. A subset (~ 15-25%) of CRPC tumours gain neuroendocrine-like phenotypes (i.e. neuroendocrine prostate cancer, NEPC), which is characterised by reduced AR expression/activity and elevated expression of neuroendocrine, mesenchymal and stem cell markers [4–6]. NEPC is associated with transcriptional and epigenetic alterations that enhance cellular plasticity (also referred to as lineage plasticity) and enable cancer cells to adaptively respond to therapy [3, 6]. The recognition of cellular plasticity as an emerging hallmark of cancer has elicited substantial efforts to identify and characterise regulators of this process [7]. Nevertheless, many aspects of this phenomenon remain poorly understood.

The ubiquitin-proteasome system (UPS) controls intracellular protein degradation and thereby regulates all cellular processes [8]. Protein turnover is balanced by the addition and removal of ubiquitin groups by ubiquitin ligases and deubiquitylating enzymes (DUBs), respectively [9]. Ubiquitin-specific peptidases (USPs) are the largest family of DUBs and play critical and diverse roles in both normal and disease states, including cancer [10]. In particular, the ubiquitin-specific peptidase USP2 has been identified as an oncogenic factor in a range of cancer types, where it enables cell proliferation and survival by stabilising key regulators of these processes [11]. Here, we expand on this earlier work by demonstrating the relevance of USP2 as a mediator of prostate cancer progression. More specifically, our findings reveal that USP2 is induced in response to ATTs and can mediate alterations to the proteome and transcriptome that are associated with aggressive neuroendocrine-like and therapy-resistant cell states. Targeting USP2 with a small molecule inhibitor effectively blocked the growth of AR-null prostate tumours. These findings justify further mechanistic and therapeutic investigation of USP2 in aggressive prostate cancer.

## Methods

### Cell line models

Prostate cancer cell lines LNCaP, VCaP, 22Rv1 and PC3 were obtained from the American Type Culture Collection (ATCC; Rockville, MD, USA). The castration-resistant LNCaP derivatives V16D, MR49F^ENZR^ and MR42D^ENZR^ were a kind gift from Prof. Amina Zoubeidi [12]. LNCaP, VCaP, 22Rv1, PC3, V16D and MR49F^ENZR^ were cultured as described previously [13]. MR42D^ENZR^ cells were cultured in RPMI 1640 medium supplemented with 10% fetal bovine serum (FBS) and 10 µM enzalutamide. All cell lines were authenticated using short tandem repeat profiling in 2026 by GNOMIX Inc or in 2018 from CellBank Australia and underwent regular testing for mycoplasma contamination.

Murine prostate cancer cell lines PBCre4:Ptenf/f KO (SKO), PBCre4:Ptenf/f:Rb1f/f KO (DKO) and PBCre4:Ptenf/f:Rb1f/f:Trp53f/f KO (TKO) were generated from previously described genetically-engineered mouse models (GEMMs) [14]. SKO, DKO and TKO cell lines were cultured in high-glucose DMEM and 10% FBS and underwent regular genotyping and testing for mycoplasma contamination.

### RNA-sequencing of patient-derived explants

RNA-sequencing data from patient-derived explants (PDE) was generated in an earlier study from our team [15] (GSE180628). Briefly, prostate cancer tissues in the form of 8 mm biopsy cores were obtained with written informed consent through the Australian Prostate Cancer BioResource from men undergoing radical prostatectomy. Ethical approval for the use of human prostate tumours was obtained from the Human Research Ethics Committees of the University of Adelaide (Adelaide, Australia; approval #H-2012-016) and St Andrew’s Hospital (Adelaide, Australia; approval #80) and all experiments were performed in accordance with the National Health and Medical Research Council of Australia (NHMRC) guidelines. Tissues were cut into ~1 mm^3^ pieces and cultured in the presence of 10 µM enzalutamide or vehicle control for 48 h according to an established protocol [16]. Total RNA was extracted using RNeasy mini kits (Qiagen) according to the manufacturer’s instructions and subjected to total RNA sequencing [15]. In this study, we examined RNA sequencing data from PDEs that exhibited a reduction in Ki67 staining in response to enzalutamide treatment (*n* = 12 tumours). Differentially expressed genes were identified as described [15]. RNA-seq data was further analysed using Gene Set Enrichment Analysis (GSEA) [17], which was done using GSEA v4.4.0 for Windows with default parameters and normalised counts as input data.

### Analysis of public transcriptomic data

Transcriptomic data were downloaded from GEO (GSE48403 [18], GSE5901 [19], GSE66187 [20], GSE230282 [21], GSE90891 [14]), cBioportal [22] (TCGA [23]) or GitHub (SU2C [24]). The Bluemn dataset [25] was kindly provided by Peter Nelson and Ilsa Coleman (Fred Hutchinson Cancer Research Center, Seattle, USA). The Beltran [26] dataset was obtained from dbGaP (accession number phs000909). The MURAL PDX dataset [27] was kindly provided by Mitchell Lawrence and Renea Taylor (Monash University, Melbourne, Australia). GSE230282 was analysed using Seurat [28] in R (v.4.4.2; https://www.R-project.org/). A summary of these transcriptomic datasets is provided in Table S1.

### Lentiviral transduction of cells

To generate LNCaP cells stably overexpressing USP2, the *USP2* (*USP2a*) open reading frame with a sequence encoding a triple FLAG tag at the 5’ end was cloned into the pJS64 lentiviral vector using Gateway cloning. A cDNA encoding mNeonGreen was also cloned into the same vector for use as a control. Lentivirus particles were prepared using a second-generation packaging system in HEK293T cells by transfection of cells with packaging vectors and pJS64 vectors, as described previously [29]. LNCaP cells were transduced with concentrated lentivirus at a multiplicity of infection of 1, followed by 2 weeks of hygromycin selection to generate LNCaP-USP2 and LNCaP-Control cells. USP2 overexpression was confirmed by immunoblotting.

### RNA sequencing of cell lines

To measure the transcriptome in LNCaP-USP2 and LNCaP-Control models, cells were seeded in full serum media in 6-well plates. Three biological replicates from consecutive cell passages, each of which were a pool of 3 technical replicates from that passage, were processed for RNA sequencing. Total RNA was extracted using a RNeasy mini kit (Qiagen) according to the manufacturer’s instructions. RNA sequencing was done by the South Australian Genomic Centre (SAGC). Sequencing libraries were constructed according to the Nugen Universal Plus mRNA-seq protocol and include 15 cycles of amplification. Equimolar pools were prepared and converted to MGI-compatible libraries using the MGIEasy Universal Library Conversion kit (MGI). Libraries were sequenced (single-end) on an MGI DNBSEQ-G400 large Flow cell platform. Resulting FASTQ files, averaging 75 million reads per sample, were analysed and quality checked using FastQC (http://www.bioinformatics.babraham.ac.uk/projects/fastqc). Reads were mapped against the human reference genome (hg38) using the STAR spliced alignment algorithm [30] (version 2.5.2b with default parameters). The reads were assigned to each exon region based on the human genome annotation from GENCODE (https://www.gencodegenes.org/) and assigned reads were summarised as counts using the featureCounts (v.1.6.4) tool [31]. The counts were normalised and PCA plots generated using the pcaExplorer package [32] in R (v.4.2; https://www.R-project.org/). Differential gene expression analysis was conducted using DESeq2 [33] in R. GSEA was done as for the PDE RNA-seq data and visualised using ggplot2 [34] in R (v.4.1.0).

### Quantitative real-time PCR

RNA from MURAL PDXs [27, 35] was extracted in a previous study [36]. Total RNA was extracted from prostate cancer cell lines using TRI Reagent (Sigma) as described previously [36] or RNeasy mini kits (Qiagen) according to the manufacturer’s instructions. Total RNA was treated with Turbo DNA-free kit (Invitrogen) and reverse transcribed using an iScript Reverse Transcriptase Supermix kit (Bio-Rad). qRT-PCR was performed in triplicate as described [36]. *GAPDH* or *ACTB* were used for normalisation of qRT-PCR data. Relative gene expression was calculated using the comparative Ct method. Primer sequences were: *USP2* (Forward: CTGCCCTGAATACCTGGTCG; Reverse: TCGGTAGGTTGGGCTGATGAT); *GAPDH* (Forward: TGCACCACCAACTGCTTAGC; Reverse: GGCATGGACTGTGGTCATGAG); *ACTB* (Forward: GGCCAACCGCGAGAA; Reverse: ATCACGATGCCAGTGGTACG); *KLK3* (Forward: ACCAGAGGAGTTCTTGACCCCAAA; Reverse: CCCCAGAATCACCCGAGCAG).

### Immunoblotting

Protein lysates from cell lines were prepared using radioimmunoprecipitation assay (RIPA) buffer containing cOmplete mini protease inhibitor (Sigma). Protein lysates from xenograft tissues were prepared by transferring frozen tissue into RIPA buffer, homogenisation using a Precellys homogeniser (6500 rpm, 3 cycles of 30 s on and 30 s off) and centrifugation at 16,100 × *g* for 10 min at 4 °C. The protein concentration of lysates was determined using Bradford assays and immunoblotting was performed as described previously [37]. Antibodies used in this study were: anti-FLAG (Sigma F1804; 1:2000), anti-Aurora A (CST D3E4Q; 1:1000); anti-Cyclin D1 (DAKO M3642; 1:1000); anti-FASN (Santa Cruz SC-20140; 1:1000; anti-GAPDH (Merck MAB374, 1:2000); anti-PGRN (Abcam ab208777, 1:1000); and anti-PLK-1 (Santa Cruz sc-17783, 1:500).

### Growth and death assays in human cell lines

Cell viability was assessed using trypan blue dye exclusion assays. Alternatively, viable and dead cells were quantified by staining cells with Annexin V-PE and 7-aminoactinomycin D (7AAD), with analysis by flow cytometry, as described previously [13]. Colony formation assays were carried out as described previously [38], except that 800 cells were seeded per well.

### Growth and death assays in murine cell lines

For drug sensitivity assays, 500 cells/well (SKO, DKO) or 350 cells/well (TKO) were seeded into 96-well plates and treated with the indicated concentrations of ML364 for 72 h. Cell growth was assessed using the CellTiter-Glo (Promega G7572) assay according to the manufacturer’s instructions. Luminescent signal was detected using a SpectraMax iD3 reader with a 0.5-second measuring time. Values were normalised to the DMSO treated sample within each cell line. Graphs shown are representative of 3 independent experiments. The IC_50_ inhibitor response was calculated using GraphPad Prism using the Variable Slope (four parameters) [inhibitor] v. response function. Cell viability/death of the SKO, DKO and TKO cells following 72 hour treatment with 10 µM ML364 was determined by staining with Annexin V APC and propidium iodide (PI), with analysis by flow cytometry.

### USP2 siRNA-mediated knockdown

Short interfering RNAs (siRNAs) targeting human *USP2* were obtained from Millennium Science and transfected into prostate cancer cells at a final concentration of 2 or 20 nM using RNAiMAX Transfection Reagent (Life Technologies) according to the manufacturer’s instructions. The sequences of the *USP2* siRNAs were: 5’-UAGUUCUCCAGGUAGUCGA-3’ and 5’-AUUCUGUGUAGCGCUUCAG-3’. AllStars Negative Control siRNA (Qiagen) was transfected at matching concentrations.

### Animal experiments

All animal procedures were approved by the SAHMRI Animal Ethics Committee (approval number SAM-21-042) and conducted in accordance with NHMRC guidelines. Luciferase-tagged PC3 cells [39] were injected intraprostatically into 8-week-old male NOD-*scid* IL2rγ^null^ (NSG) mice (1 × 10^6^ cells in 10 µL of PBS per mouse). After a period of 10 days for tumour establishment, mice were given daily intraperitoneal injections of 30 mg/kg ML364 or vehicle control. Before commencing treatment, mice were randomised into groups; the absence of a significant difference in baseline tumour volume between groups was confirmed using an independent sample t-test (*p* > 0.05). The investigators were not blinded to group allocation. Sample sizes (10 mice per group) were estimated as described previously [40], with an effect size of 0.7, a statistical power level of 0.8 and a significance level of 0.05. Whole-body imaging to monitor luciferase-expressing PC3 cells was performed weekly using an IVIS Spectrum in vivo Imaging System (PerkinElmer). D-luciferin (potassium salt, PerkinElmer) was dissolved in sterile deionised water (0.03 g/mL) and injected subcutaneously (3 mg/20 g of mouse body weight) before imaging. Bioluminescence was reported as the sum of detected photons per second from a constant region of interest. Near the end of week 4 of treatment, one mouse in the control group and 4 in the ML364-treated group developed tumours exceeding the limits set by the approved animal ethics protocol and were humanely euthanised. After all mice were humanely euthanised after 4 weeks of treatment, tumours were removed and flash frozen in liquid nitrogen prior to immunoblotting.

### Seahorse extracellular flux analysis

To evaluate glycolysis in LNCaP cells overexpressing USP2, XF Glycolysis Stress Test Kits (103020-100; Seahorse Bioscience) were used. Cells (1.5 × 10^4^ cells/well) were seeded into Poly-D-Lysine-coated XF96 well cell culture microplates (Agilent) in standard culture media (RPMI with 10% FBS) for 24 h. One hour before beginning the assay, culture media was replaced with Seahorse XF base medium containing 2 mM glutamine. Extracellular acidification rates (ECAR) were measured in glucose-free medium before and after the sequential injection of 10 mM glucose, 1 µM oligomycin A and 50 mM 2-Deoxy-D-glucose (2DG). Results were analysed using Seahorse Wave software for XF Analysers. ECAR values were normalised to cell numbers in each well. To evaluate glycolysis in response to ML364 treatment, assays were performed as described above, except cells were seeded in drug-containing media for 24 h before initiation of the assay.

### Proteomics

MR42D^ENZR^ cells were seeded in 10 cm dishes (2 × 10^6^ cells per dish) and treated with 5 µM ML364 or vehicle control for 24 h (5 replicates of each). After the treatment period, cells were washed with PBS and then harvested in 0.7 ml of lysis buffer (50 mM Tris-HCl pH 8.0) containing 1x cOmplete mini EDTA-free protease inhibitors (Roche). Protein lysates were generated using a Dounce homogeniser and a final concentration of 1% (w/v) dodecylmaltoside was added per sample. The protein concentration of lysates was determined using EZQ protein assays (Invitrogen) and 10 μg of protein per sample was reduced with 10 mM TCEP-HCL (Millipore) in 100 mM ammonium bicarbonate (Sigma) for 30 min at 56 °C. The reduced proteins were then alkylated with 20 mM chloroacetamide (Sigma) for 30 min at room temperature. Alkylated proteins were digested with trypsin at a ratio of 1:50 (protein to trypsin) in a total volume of 200 μl overnight at 37°C, after which trypsinisation was quenched using 0.1% formic acid.

Digested peptides were cleaned up using custom C18 StageTips and eluted using 80% acetonitrile in 0.1% formic acid. Peptides were resolved using reverse-phase liquid chromatography on a Dionex Ultimate 3000 UPLC (ThermoFisher Scientific). The trap column was a PepMap™ 100 trap cartridge (0.3 ×5 mm, 5 µm C18; ThermoFisher Scientific) and the analytical column was a 40 cm in-house pulled column created from 75 µm inner diameter fused silica capillary packed with 1.9 µm ReproSil-Pur C18 beads (Dr. Maisch, Ammerbuch, Germany). Buffer A was water with 0.1% formic acid and buffer B was 79.9% acetonitrile, 20% water and 0.1% formic acid. The flow rate was 300 nl/min utilizing a separation gradient of 8 to 31.2% acetonitrile. The Orbitrap Exploris™ 480 Mass Spectrometer (Thermo Fisher Scientific) was operated in data independent mode (DIA) with a 60,000 resolution MS scan of 390-1010 m/z, AGC of 200% and max injection time of 100 ms followed by 37 30,000 resolution DIA scans of 16 m/z width from 396-1004 m/z, 33% collision energy, standard AGC target and max injection time of 40 ms. Gas phase fractionation was used to generate a spectral library from a pool of all samples. Five iterative injections of pooled peptides were analysed using m/z windows of 350-500, 490-600, 590-700, 690-800 and 890-1200 m/z, respectively. The Exploris 480 was operated in data-dependent mode (DDA) with MS1 settings: 60,000 resolution, normalised AGC target of 3e6 with auto injection time. For MS2, 15,000 resolution intensity, threshold of 3e5, dynamic exclusion time of 45 sec, AGC target 1e5 and 33% collision energy was used.

The data were processed using Spectronaut v16.0.220606.53000 using default settings and the Uniprot human database (August 2021, containing 202,160 entries). The spectral library was generated using Proteome Discoverer v2.4 SP1 and the same Uniprot database. GSEA and visualisation of proteomics data was done as described for RNA-seq. Over-representation analysis was conducted using MSigDB [41].

### Statistical analysis

Statistical analysis was performed using GraphPad Prism 7.02 software. Statistical methods are included in figure legends. Evaluation of normality was done using Kolmogorov-Smirnov tests when sample sizes were ≥5; for experiments with smaller sample sizes, normality was assumed. A sample size of *n* = 3 was used unless otherwise mentioned and all experiments were performed at least three times. No samples were excluded from analysis.

## Results

### USP2 is negatively regulated by the androgen receptor signalling axis

**Fig. 1:**
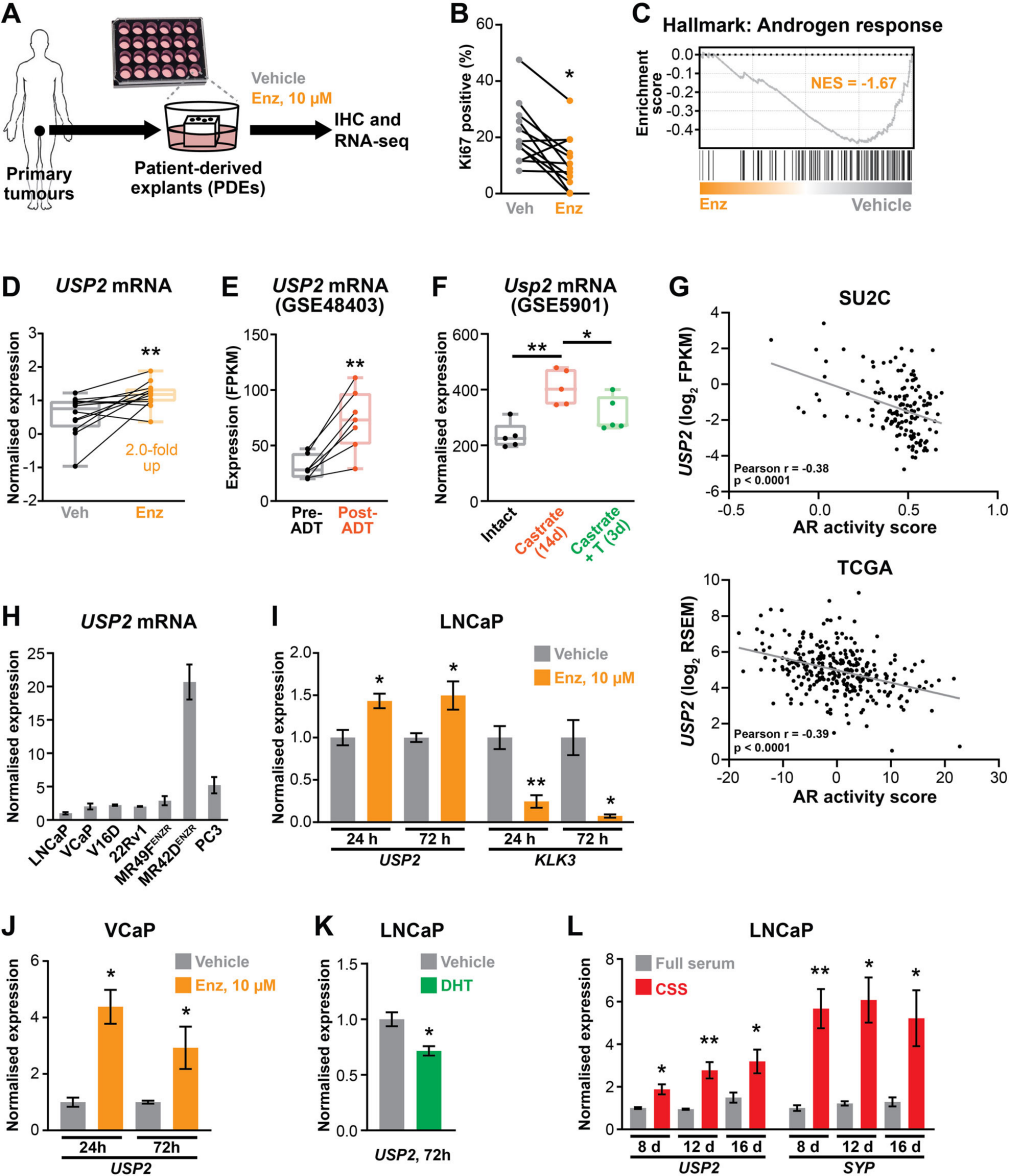
USP2 is negatively regulated by the androgen receptor signalling axis. **A** Schematic demonstrating the design of the patient-derived explant (PDE) experiment. IHC, immunohistochemistry. **B** Ki67 positivity in prostate cancer PDEs (*n* = 9 tumours) exposed to vehicle (Veh) or 10 µM enzalutamide (Enz). Groups were compared using a paired t-test. **C** Enzalutamide suppresses androgen response, as demonstrated by gene set enrichment analysis. NES, normalised enrichment score. **D** *USP2* mRNA levels in prostate cancer PDEs exposed to Veh or Enz. Groups were compared using a paired t test and average fold-change is shown. For D-E, boxes represent 25th to 75th percentiles, top and bottom lines are minimum and maximum and middle line is the median. **E** *USP2* mRNA levels in 7 prostate tumours pre- and post-androgen deprivation therapy. Groups were compared using a paired t test. FPKM, fragments per kilobase of exon per million mapped reads. **F** *Usp2* mRNA levels in mouse prostate tissues (*n* = 5 per treatment group) from intact or castrate mice or castrate mice supplemented with the androgen testosterone (T) pellet for 3 days. Groups were compared using one-way ANOVA and Tukey’s multiple comparisons tests. **G** *USP2* expression and AR activity are negatively correlated in SU2C (top) and TCGA (bottom) datasets. AR activity scores were calculated using Single Sample GSEA. P and r values were determined using Pearson’s correlation tests. **H** *USP2* mRNA levels in a panel of prostate cancer cell lines, as determined using qRT-PCR. Error bars are ± standard error of the mean (SEM). LNCaP was set to 1.*USP2* mRNA levels are elevated in response to 10 µM enzalutamide (Enz) in LNCaP (**I**) and VCaP (**J**) cells and reduced in response to DHT (**K**) in LNCaP cells. Groups were compared using unpaired t tests. Error bars are ± standard error of the mean (SEM). In **I**, an androgen-induced gene, *KLK3*, is shown as a control. **L** *USP2* mRNA levels are elevated in LNCaP cells growing in charcoal-stripped serum (CSS) compared to standard growth media (full serum). Groups were compared using unpaired t tests at each time point. Error bars are ± standard error of the mean (SEM). A gene known to be induced by androgen deprivation, *SYP*, is shown as a control. In all panels: *, *p* < 0.05; **, *p* < 0.01; ***.

The acute adaptive response of clinical prostate tumours to enzalutamide was evaluated using a prostate-derived explant (PDE) system developed by our team [16, 42]. We leveraged data from a previous experiment [15] in which PDEs were treated with 10 µM enzalutamide for 48 h, after which immunohistochemistry (IHC) and RNA-sequencing (RNA-seq) were used to evaluate molecular responses to the AR antagonist treatment (Fig. 1A). Enzalutamide caused a significant reduction in levels of a marker of proliferation, Ki67, as determined by IHC (Figs. 1B and S1). Gene set enrichment analysis (GSEA) of RNA-seq data demonstrated a decrease in AR activity in response to enzalutamide treatment, providing evidence for on-target activity of the drug (Fig. 1C). Of the 84 transcripts significantly altered with enzalutamide treatment (*p* ≤ 0.05, logFC ≥ 0.5; Table S2), *Ubiquitin Specific Peptidase 2* (*USP2*) was of interest given its emerging relevance to cancer development and progression [43]. *USP2* was upregulated approximately 2.0-fold with enzalutamide treatment (Fig. 1D), suggesting that its expression is inhibited by AR. Supporting this concept, we found that *USP2* was increased following androgen deprivation therapy in patient tumours (Fig. 1E). *Usp2* was also elevated in response to castration in mouse prostate tissues and this elevation was suppressed with subsequent androgen supplementation (Fig. 1F). Moreover, *USP2* levels were negatively correlated with AR activity in metastatic CRPC (Stand Up 2 Cancer cohort) and primary (TCGA cohort) tumours (Fig. 1G).

To experimentally investigate the relationship between AR signalling and USP2, we first measured *USP2* expression in a panel of PCa cell lines. *USP2* mRNA levels were highest in the enzalutamide-resistant, AR-positive, neuroendocrine (NE)-like model MR42D^ENZR^ [12], followed by PC3, which is AR-null and expresses some NE markers [44], and lowest in the AR-positive androgen-responsive LNCaP model (Fig. 1H). Enzalutamide increased the expression of *USP2* in LNCaP and VCaP cells (Fig. 1I, J), mirroring the findings from the PDE system. Conversely, treatment with the physiological androgen 5α-dihydrotestosterone (DHT) significantly decreased *USP2* expression in LNCaP cells (Fig. 1K). Moreover, LNCaP cells grown in charcoal-stripped serum (CSS) for extended periods to deplete androgens, an approach that mimics androgen deprivation therapy, also exhibited upregulation of *USP2* (Fig. 1L). This occurred concurrently with induction of the neuroendocrine marker *Synaptophysin* (*SYP*) (Fig. 1L), which reflects an established response of LNCaP cells to androgen deprivation whereby they gain features and markers of neuroendocrine cell states [36, 45].

### USP2 is elevated in neuroendocrine prostate cancer and associated with cell plasticity

**Fig. 2:**
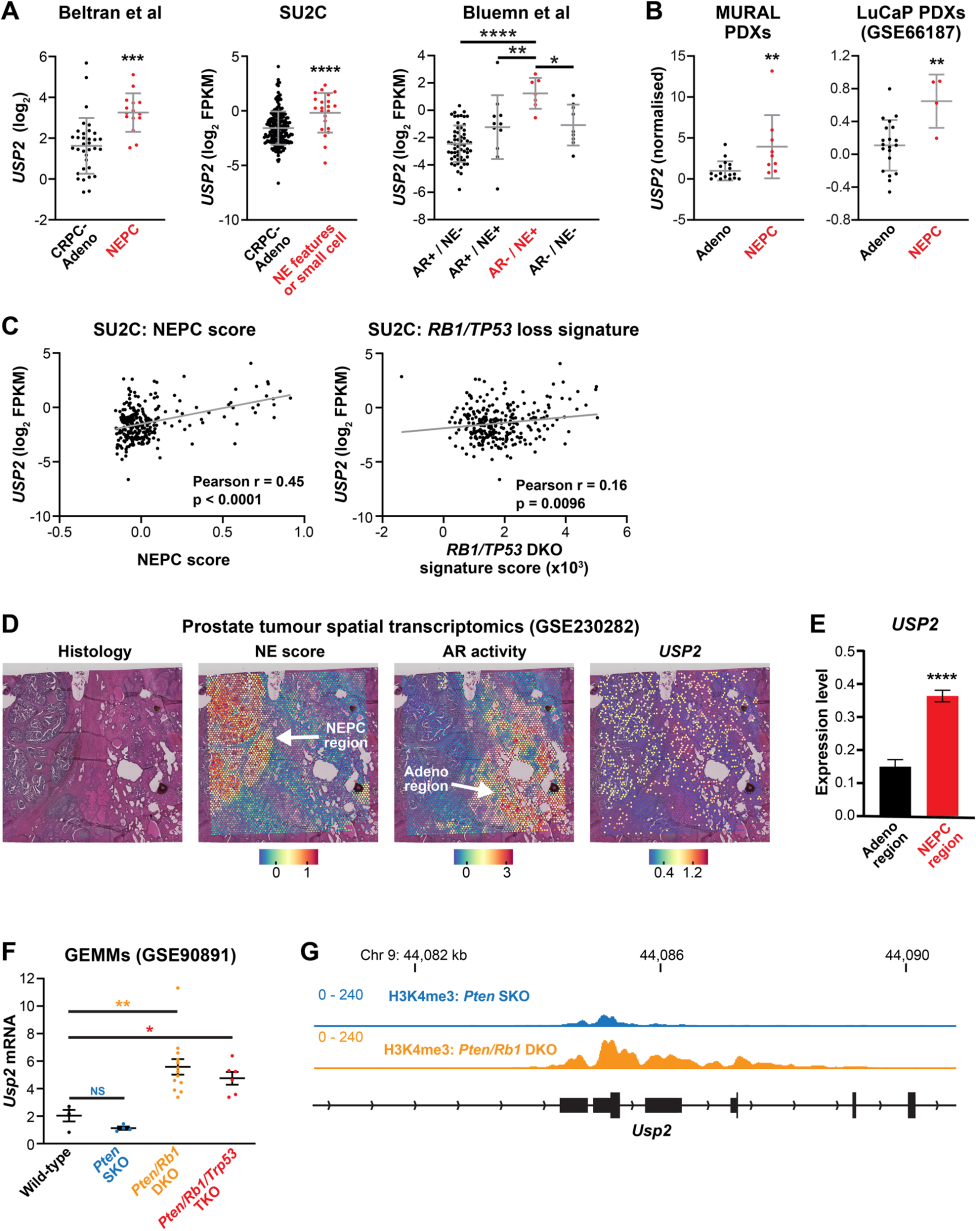
USP2 is elevated in neuroendocrine prostate cancer and associated with cell plasticity. **A** *USP2* mRNA levels are elevated in neuroendocrine prostate cancer (NEPC) or tumours with neuroendocrine (NE) features compared to CRPC-adenocarcinoma (CRPC-Adeno) in 3 distinct cohorts. Middle line is mean, top and bottom lines represent standard deviation (SD) from the mean. Groups were compared using unpaired t tests (Beltran, SU2C) or one-way ANOVA and Tukey’s multiple comparisons tests (Bluemn). **B** *USP2* mRNA levels are elevated in NEPC PDXs compared to adenocarcinoma (Adeno) PDXs in 2 distinct cohorts. The middle line is the mean, and the top and bottom lines represent the SD from the mean. Groups were compared using unpaired t tests. **C** *USP2* expression is positively correlated with gene signatures of NEPC (left) and *RB1*/*TP53* double knockout (DKO) (right). Gene signature scores were calculated using Single Sample GSEA. P and r values were determined using Pearson’s correlation tests. **D** Spatial transcriptomics (GSE230282) of a prostate tumour exhibiting distinct NE- and AR-enriched regions, which represent NEPC and adenocarcinoma (Adeno), respectively. The right panel shows *USP2* expression within the tumour. **E** Quantitation of *USP2* expression from spatial transcriptomic data of the tumour shown in (**D**). The expression of *USP2* was compared in regions of NEPC or AR-driven adenocarcinoma (Adeno). Groups were compared using an unpaired t test. **F** *Usp2* expression in prostate tissues from genetically-engineered mouse models (GEMMs) with the indicated genotypes. Middle line is mean, top and bottom lines represent SD from the mean. Groups were compared using ANOVA and Tukey’s multiple comparisons tests. **G** H3K4me3 ChIP-seq signal proximal to the *Usp2* gene in prostate tissues from GEMMs with the indicated genotypes. In all panels: *, *p* < 0.05; **, *p* < 0.01; ***, *p* < 0.001; ****, *p* < 0.0001.

Given the inverse relationship between USP2 expression and AR activity, we hypothesised that USP2 would be elevated in advanced PCa disease states characterised by loss of AR expression/activity. Indeed, we observed that USP2 levels were higher in NEPC compared to CRPC-adenocarcinoma in multiple clinical datasets (Fig. 2A) [24, 25, 46]. A similar result was observed in the MURAL and LuCaP PDX collections (Fig. 2B) [27, 47]. In line with these observations, the expression of *USP2* in metastatic CRPC was positively correlated with an NEPC phenotype score (Fig. 2C), which is derived from a well-characterised NEPC gene set [26]. Combined *RB1*/*TP53* loss is known to promote cell plasticity and the emergence of neuroendocrine-like phenotypes [3]; accordingly, *USP2* expression was also correlated with a *RB1*/*TP53* loss transcriptomic signature (Fig. 2C) [48]. Evaluation of a larger panel of gene sets reinforced the positive association between *USP2* and NEPC and also revealed that *USP2* was correlated with epithelial-mesenchymal transition (EMT) (Fig. S2A), another manifestation of cancer cell plasticity that is a feature of NEPC tumours [3]. These associations were also evident in primary prostate cancer (Fig. S2B). Despite being associated with aggressive tumour features, *USP2* mRNA levels were not predictive of disease progression or overall survival in the TCGA and SU2C cohorts, respectively (Fig. S2C).

To visualise *USP2* expression in prostate cancer, we explored a spatial transcriptomic dataset from a tumour reported to comprise distinct regions of NEPC and AR-driven adenocarcinoma [21]. Our own analysis of this tumour confirmed the co-existence of these spatially separate cancer phenotypes (Fig. 2D) and demonstrated that USP2 was more highly expressed in cells with NEPC features (Fig. 2D, E).

We next analysed expression of the murine *Usp2* gene in genetically-engineered mouse models (GEMMs) that are representative of aggressive and highly plastic prostate cancer states. More specifically, tumours with double knockout (DKO) of *Pten*/*Rb1* and triple knockout (TKO) of *Pten*/*Rb1*/*Trp53* exhibit lineage plasticity and resistance to AR-targeted therapies whereas single knockout (SKO) of *Pten* does not induce such phenotypes [14]. Paralleling the human findings, *Usp2* was upregulated in tumours with the DKO and TKO tumours compared to wild-type or SKO tissues (Fig. 2F). Supporting the RNA expression data, the promoter of the *Usp2* gene exhibited elevated levels of a histone mark associated with active transcription, H3K4me3, in mouse prostate tissues with *Pten*/*Rb1* loss (Fig. 2G). Collectively, these results demonstrate a robust association between USP2 and aggressive AR-independent disease phenotypes, most notably NEPC.

### USP2 drives the emergence of a therapy-resistant and plastic disease state

**Fig. 3:**
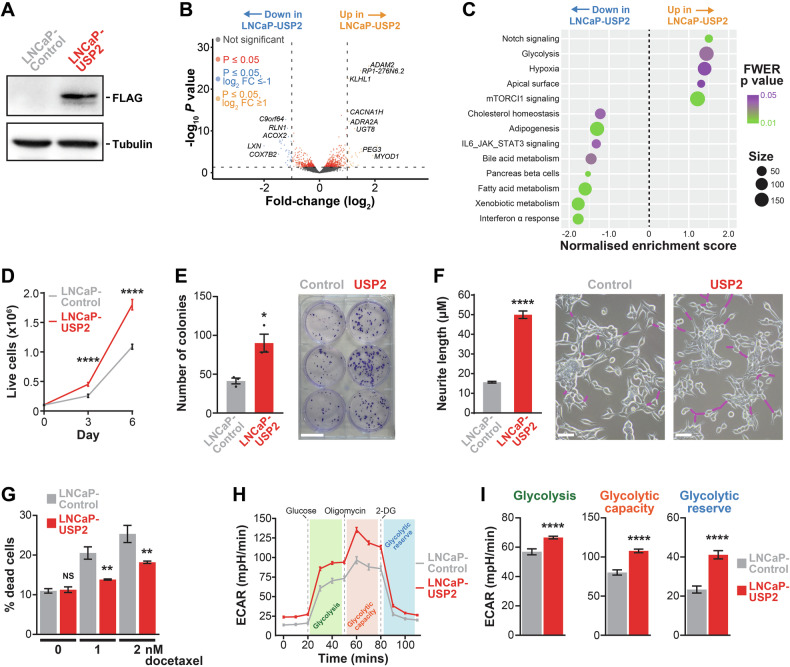
USP2 drives the emergence of a therapy-resistant, neuroendocrine-like disease state. **A** Immunoblotting showing overexpression of USP2 in stably transduced LNCaP cells. Tubulin was used as a loading control. **B** Volcano plot summarising differential gene expression analysis of LNCaP-USP2 versus LNCaP-Control cells. Select genes are shown. **C** Hallmark gene sets associated with USP2 overexpression, as determined by gene set enrichment analysis. FWER, family-wise error rate. **D** Overexpression of USP2 promotes the growth of LNCaP cells, as evaluated by trypan blue growth assays. **E** Overexpression of USP2 promotes clonogenic survival of LNCaP cells, as evaluated by colony formation assay. Representative images are shown on the right (scale bar = 2 cm). **F** Neurite length is elevated in LNCaP cells overexpressing USP2. Representative phase contrast images are shown on the right, with neurite outgrowths traced in magenta (scale bars = 25 μm). **G** Cell death (% of total cell number) in LNCaP-Control and LNCaP-USP2 cells after 3 days of treatment with the indicated doses of docetaxel. Cell death was determined by the trypan blue assay. **H** Overexpression of USP2 increases glycolysis in LNCaP cells. Glucose-starved cells were treated with 2 g/L D-glucose, 1 μM oligomycin and 100 mM 2-Deoxyglucose (2-DG) at the indicated timepoints and extracellular acidification rates (ECAR; normalised to cell number) were calculated every 10 min (see Methods for more information). **I** Representative quantification of glycolytic function (measured following the addition of D-glucose), glycolytic capacity (measured following the addition of oligomycin) and glycolytic reserve (measured following the addition of 2-DG). For (**D–I**), data are shown as mean ± SEM. For (**D–G**) and **I**, LNCaP-USP2 and LNCaP-Control cells were compared using unpaired t tests. In all panels: *, *p* < 0.05; **, *p* < 0.01; ***, *p* < 0.001; ****, *p* < 0.0001.

To determine whether USP2 can directly influence prostate cancer phenotype, we generated a LNCaP derivative overexpressing FLAG-tagged USP2 using a lentiviral-based approach. LNCaP was chosen because it has low endogenous levels of USP2 and is a well-studied, AR-responsive model of prostate adenocarcinoma. USP2 overexpression in the resultant model (referred to as LNCaP-USP2) was confirmed by immunoblotting (Figs. 3A and S3), compared to cells transduced to express an mNeonGreen control gene (referred to as LNCaP-Control). RNA-sequencing was used to further explore the impact of USP2 overexpression on the LNCaP phenotype. We identified 345 genes that were differentially expressed (*p* < 0.01) between LNCaP-USP2 and LNCaP-Control cells (Fig. 3B and Table S3). GSEA revealed that USP2 overexpression was positively associated with Notch signalling, glycolysis and hypoxia (Fig. 3C), pathways that have been linked to prostate cancer therapy resistance and cell plasticity [3]. Conversely, USP2 overexpression was negatively associated with interferon signalling and metabolism of xenobiotics, fatty acids and bile acids (Fig. 3C).

We next evaluated the phenotypic effects of USP2 overexpression. LNCaP-USP2 cells grew more quickly than control cells in standard media (Fig. 3D) and had significantly enhanced colony-forming ability (Fig. 3E). Moreover, we noted that LNCaP-USP2 cells exhibited an increase in the number and length of neurites (Fig. 3F), a phenotype that has been associated with epithelial-neuroendocrine switching [49]. Since USP2 was identified as being induced by enzalutamide and elevated in aggressive, AR-independent therapy-resistant tumours, we evaluated whether its overexpression influenced response to prostate cancer therapies. LNCaP-USP2 cells grew approximately 2-fold faster than LNCaP-Control cells in androgen-depleted (i.e. charcoal-stripped) serum (Fig. S4A), which mimics androgen deprivation in patients. Moreover, LNCaP-USP2 cells were less sensitive to the taxane chemotherapeutic docetaxel—a major treatment modality for advanced prostate cancer [50]—than the control line (Fig. 3G). Interestingly, despite eliciting a growth advantage in response to androgen deprivation and chemotherapy, USP2 overexpression did not confer resistance to enzalutamide in this context (Fig. S4B).

Elevated glycolysis is a distinguishing feature of NEPC [51]. Since one of the most strongly upregulated pathways in LNCaP-USP2 cells was glycolysis and these cells gained other features of NEPC such as neurite outgrowths, we further investigated the metabolic alterations induced by USP2 overexpression. More specifically, we employed extracellular flux analysis of live cells using a Seahorse platform. Supporting the transcriptomic analyses, we observed that LNCaP-USP2 cells exhibited a significantly greater extracellular acidification rate (ECAR), a proxy for glycolysis, compared to LNCaP-Control cells (Fig. 3H, I).

Collectively, these assays reveal that upregulation of USP2 promotes the emergence of aggressive oncogenic phenotypes that modify cell metabolism and elicit resistance to androgen deprivation and chemotherapy.

### USP2 stabilises key oncoproteins in prostate cancer cells

**Fig. 4:**
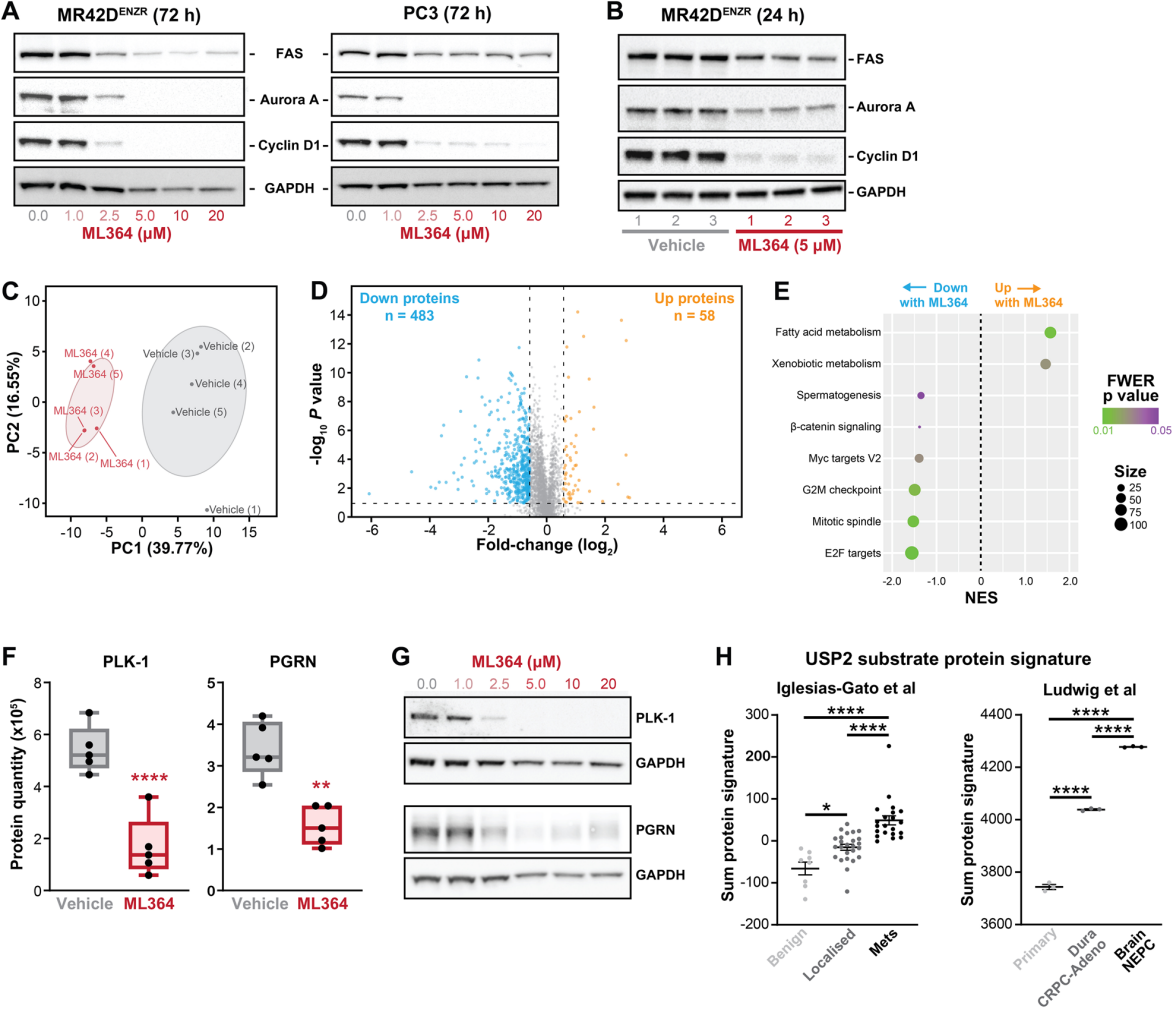
Proteomics identifies putative USP2 substrates and mediators of prostate cancer progression. **A** Immunoblotting showing a dose-dependent loss of known USP2 substrates (FAS, Aurora A and cyclin D1) in MR42D^ENZR^ or PC3 cells. GAPDH was used as a loading control. **B** Immunoblotting showing loss of FAS, Aurora A and cyclin D1 in MR42D^ENZR^ cells treated with 5 µM ML364 for 24 h. GAPDH was used as a loading control. **C** Principal component analysis (PCA) of total proteomic data following treatment of MR42D^ENZR^ cells treated with ML364. **D** Volcano plot illustrating differentially abundant proteins in response to ML364. Proteins with lower abundance (logFC ≤ 0.5, *p* < 0.05) and higher abundance (logFC ≥ 0.5, *p* < 0.05) are shown in blue and orange, respectively. **E** Hallmark gene sets associated with ML364 treatment, as determined by gene set enrichment analysis. FWER, family-wise error rate; NES, normalised enrichment score. **F** Quantities of Polo-like kinase 1 (PLK-1) and progranulin (PGRN) according to mass spectrometry-based proteomics. Groups were compared using unpaired t tests. **G** Immunoblotting showing a dose-dependent loss of known USP2-dependent proteins PLK-1 and PGRN in MR42D^ENZR^ cells. GAPDH was used as a loading control. **H** Expression of a USP2 substrate protein signature in two different clinical proteomic datasets [60, 61]. The Iglesias-Gato cohort is comprised of benign samples (*n* = 8), localised prostate tumours (*n* = 28) and metastatic tumours (*n* = 22). The Ludwig cohort is comprised of 3 tumours from the same patient: primary tumour, an adenocarcinoma CRPC dura metastasis (CRPC-Adeno) and a neuroendocrine CRPC brain metastasis (NEPC) (3 technical replicates of each). Datapoints represent sums of 422 putative USP2 substrates in each sample. Groups were compared using one-way ANOVA and Tukey’s multiple comparisons tests. In all panels: *, *p* < 0.05; **, *p* < 0.01; ***, *p* < 0.001; ****, *p* < 0.0001.

To provide insights into the oncogenic functions of USP2 in prostate cancer, we undertook studies to characterise its substrates, taking advantage of a small molecule, highly selective USP2 inhibitor, ML364 [52]. First, we confirmed that known USP2 substrates with prominent roles in cancer growth and progression— Aurora kinase A (Aurora A) [53], fatty acid synthase (FAS) [54] and cyclin D1 [55]—were destabilized following ML364 treatment. The levels of these proteins were markedly and dose-dependently reduced by ML364 in MR42D^ENZR^ and PC3 prostate cancer cells over a period of 3 days (Fig. 4A). Having validated the activity and specificity of ML364, we then measured the effects of this inhibitor at a whole-proteome level using mass spectrometry. For this experiment, the MR42D^ENZR^ model was used because it exhibited the highest USP2 expression (Fig. 1H). To facilitate the identification of direct USP2 substrates as opposed to proteins that may be altered via downstream effects, MR42D^ENZR^ cells were treated for just 24 h with 5 µM ML364; we confirmed that known USP2 substrates were reduced by this acute treatment (Fig. 4B). Subsequently, the proteins from ML364- and vehicle-treated cells were subjected to analysis by mass spectrometry. Principal component analysis revealed that ML364 had a robust effect on the proteome (Fig. 4C), with 541 proteins being significantly altered (FDR *q* value ≤ 0.05 and fold change ≥ 1.5) compared to the control treatment (Fig. 4D). Of these proteins, the vast majority (89%; 483/541) were downregulated (Fig. 4D and Table S4), which may reflect the function of USP2 in promoting protein stability via deubiquitination. GSEA revealed that the pathways most strongly inhibited by ML364 were related to the cell cycle, including E2F targets, G2M checkpoint and mitotic spindle (Fig. 4E). These findings were corroborated by over-representation pathway analysis of the 483 significantly downregulated proteins, which revealed enrichment of cell cycle pathways as well as glycolysis (Table S5). Conversely, ML364 treatment led to elevated expression of proteins involved in fatty acid and xenobiotic metabolism (Fig. 4E); this latter observation was consistent with our transcriptomic data, in which we noted that these pathways were suppressed in cells overexpressing USP2 (Fig. 3C).

To further validate the proteomic data, we further investigated two proteins—Progranulin (PGRN, encoded by the *GRN* gene) and Polo-like kinase 1 (PLK-1)—that were downregulated in response to ML364 treatment according to the proteomics experiment (Fig. 4F). Both of these factors are oncoproteins with reported roles in cancer cell survival and resistance to chemotherapy [56, 57]. Moreover, PLK-1 has been linked to lineage plasticity in prostate cancer and is considered a viable therapeutic target in NEPC [58, 59]. Immunoblotting demonstrated that PGRN and PLK-1 were reduced in a dose-dependent manner by ML364 in MR42D^ENZR^ cells (Fig. 4G). To further explore the relevance of our proteomic data to clinical prostate cancer, the protein levels of putative USP2 substrates (i.e. proteins downregulated by ML364 in MR42D^ENZR^ cells) were evaluated in a proteomic dataset obtained from benign tissues and primary and metastatic tumours [60]. We observed an overall increase in abundance of its putative substrates in metastatic tumours compared to localised tumours or benign tissues (Fig. 4H). In a distinct set of primary and metastatic tumour tissues from a single patient [61], the abundance of USP2’s putative substrates was elevated in the metastases and highest in a brain lesion with NEPC histology (Fig. 4H). Collectively, these findings demonstrate that USP2 stabilises an oncogenic proteome that is linked to survival, therapy resistance and plasticity of prostate cancer cells.

### Inhibiting USP2 as a therapeutic strategy for aggressive prostate cancer

**Fig. 5:**
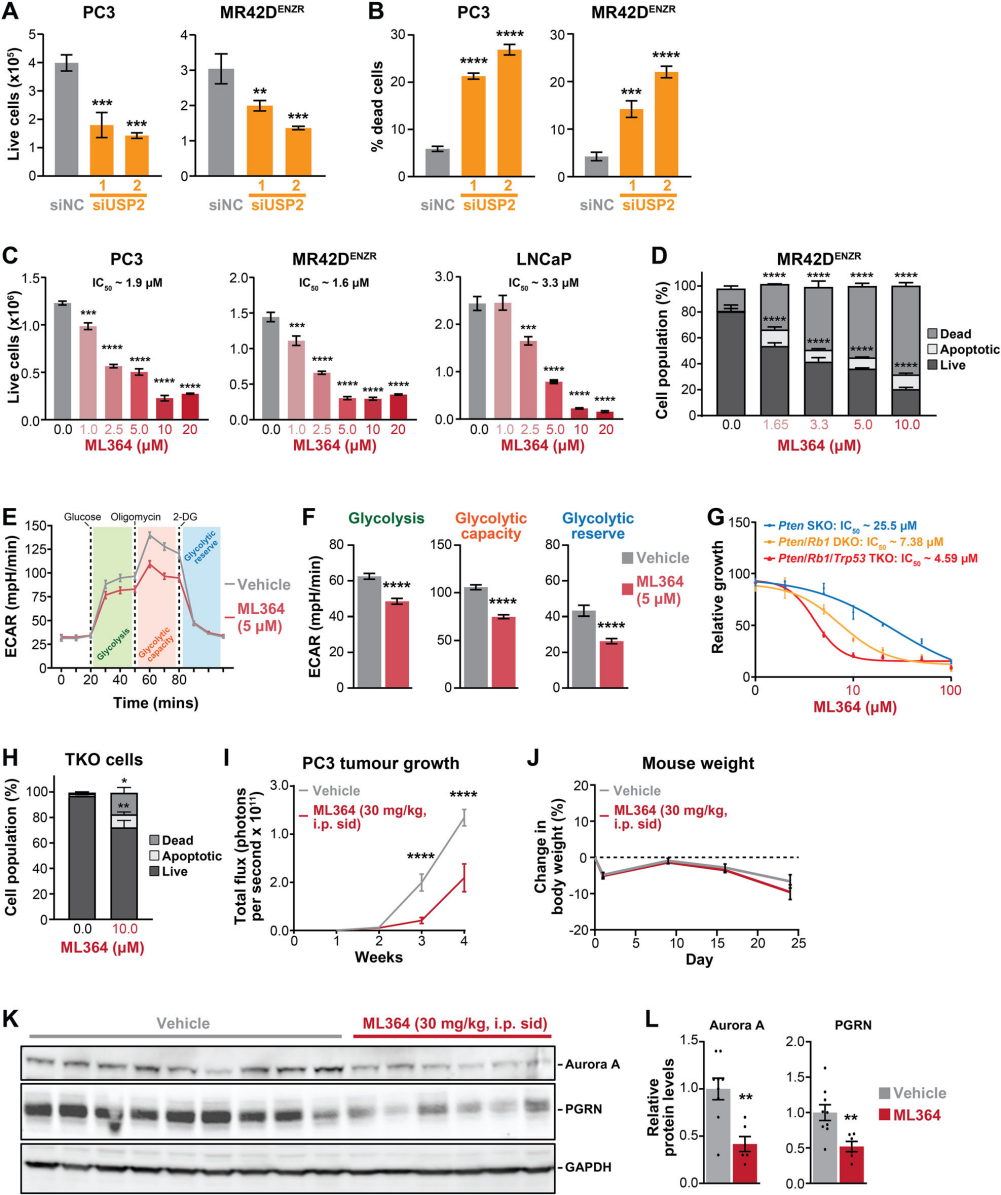
Inhibiting USP2 as a therapeutic strategy for aggressive prostate cancer. Knockdown of USP2 inhibits cell growth (**A**) and induces cell death (**B**). PC3 and MR42D^ENZR^ cells were transfected with 2 distinct USP2-targeting siRNAs and trypan blue growth assays were performed. Data are representative of three independent experiments. Groups were compared using ANOVA and Dunnett’s multiple comparison tests. **C** Treatment of prostate cancer cells with ML364 decreased growth of prostate cancer cells, as determined using trypan blue growth assays. Live cells were counted 3 days after treatment. Data are representative of three independent experiments. Groups were compared using ANOVA and Dunnett’s multiple comparison tests. **D** Treatment with ML364 caused apoptosis of MR42D^ENZR^ prostate cancer cells, as determined by flow cytometric assessment of Annexin V-PE binding and 7-AAD influx. Bars represent SEM. Dead and apoptotic cell numbers were compared between groups using ANOVA and Tukey’s multiple comparison tests. **E** Inhibiting USP2 decreases glycolysis in LNCaP cells. Extracellular acidification rates (ECAR) were calculated as described in Fig. 3H and Methods. **F** Representative quantification of glycolytic function (measured following the addition of D-glucose), glycolytic capacity (measured following the addition of oligomycin) and glycolytic reserve (measured following the addition of 2-DG). Groups were compared using unpaired t tests. **G** Impact of ML364 on the growth of genetically engineered mouse models with the indicated genotypes using a full dose curve from 0 to 100 μM. **H** ML364 causes apoptosis of *Pten*/*Rb1*/*Tp53* triple knockout (TKO) cells, as determined by flow cytometry. Dead and apoptotic cell numbers were compared as in (**D**). **I** ML364 inhibits growth of PC3 xenografts. The data shows luciferase intensity as assessed by whole-animal bioluminescent imaging over time in mice injected with PC3 cells that were treated with 30 mg/kg ML364 (intraperitoneally daily (i.p. sid)) or vehicle control. Treatment effects were evaluated using unpaired two-sided t tests. **J** Effect of ML364 on the weight of mice harbouring PC3 xenografts over the treatment period. No significant difference in mouse weight between the ML362 and vehicle groups was observed at any time point. **K** Immunoblotting showing loss of Aurora A and PGRN proteins in PC3 xenografts treated with ML364. GAPDH was used as a loading control. **L** Quantitation of Aurora A and PGRN protein levels from (**K**). Levels of both proteins were normalised to GAPDH and the vehicle group was set to 1. Treatment effects were evaluated using unpaired two-sided t tests. In all panels, data shown are the mean ± SEM and: *, *p* < 0.05; **, *p* < 0.01; ***, *p* < 0.001; ****, *p* < 0.0001.

Our previous findings revealed USP2 to be a candidate driver of prostate cancer growth and therapy resistance, particularly in aggressive variant disease states, by stabilising key oncoproteins. To address its potential as a therapeutic target, we first tested the effects of USP2 knockdown using siRNAs. Two distinct siRNAs were evaluated, both of which effectively reduced *USP2* mRNA levels by ~80–90% in PC3 and MR42D^ENZR^ cells (Fig. S5A) and reduced the protein levels of the USP2 substrate Aurora A (Fig. S5B) when transfected at a concentration of 20 nM. Importantly, knockdown of USP2 also reduced growth and potently induced death of PC3 and MR42D^ENZR^ cells (Fig. 5A, B). We also employed a lower siRNA concentration (2 nM) that was more tolerable to prostate cancer cells to investigate whether targeting USP2 influenced response to chemotherapy. This experiment revealed that USP2 knockdown sensitised PC3 cells to treatment with sub-effective doses of docetaxel (Fig. S5C, D). Collectively, these findings further validate the role of USP2 in promoting prostate cancer cell survival and therapy resistance.

Subsequently, we evaluated the potential therapeutic utility of ML364. Using trypan blue assays, we found that ML364 inhibited the growth of MR42D^ENZR^, PC3 and LNCaP cells with IC_50_ values of 1.6, 1.9 and 3.3 µM, respectively (Fig. 5C). Cell death elicited by ML364 occurred by apoptosis, as demonstrated using flow cytometric analysis of Annexin V-PE and 7-aminoactiomycin D (7-AAD) (Fig. 5D). As another readout of anti-cancer activity, we used Seahorse assays to evaluate cellular metabolism and found that ML364 caused a significant suppression of glycolysis (Fig. 5E, F), an observation that was in agreement with our findings from the USP2-OE model and the proteomics data and reinforced USP2’s role in glycolysis.

The observation that *Usp2* may also play a role in murine prostate cancer progression and cell plasticity (Fig. 2D) led us to test ML364 in cell lines derived from GEMMs lacking tumour suppressor genes. Growth assays revealed that cells lacking only *Pten* (SKO) were the least sensitive, whereas cells lacking *Pten*/*Rb1* (DKO) or all 3 genes (TKO) were more sensitive to ML364 (Fig. 5G). Cell death in GEMM cells was via apoptosis (Fig. 5H), paralleling the findings from the human models.

Given its efficacy against in vitro prostate cancer models, we assessed ML364 in vivo. Luciferase-labelled PC3 cells were used to establish orthotopic xenografts in NSG mice. After tumour establishment, mice were treated daily with 30 mg/kg ML364 (intraperitoneally) or vehicle control. Weekly monitoring using whole-animal bioluminescence imaging revealed that ML364 significantly reduced growth of this rapidly growing tumour model (Fig. 5I). Over the course of the experiment, treatment with ML364 did not cause mice to lose weight (Fig. 5J) and no other signs of overt toxicity were noted. Analysis of tumour protein by immunoblotting revealed a reduction in the protein levels of Aurora A and PGRN (Fig. 5K, L), substantiating the on-target activity of ML364. Collectively, these results demonstrate that USP2 is a viable therapeutic target in prostate cancer and justify further development of ML364 as a drug candidate.

## Discussion

The onset of CRPC is frequently associated with enhanced lineage plasticity and the acquisition of new phenotypes, including transitions to neuroendocrine, stem-like and mesenchymal cell states [3, 7]. Most regulators of prostate cancer cell plasticity identified to date are transcription factors and chromatin-modifying enzymes [3, 7]. Here, we highlight the importance of another mechanism by which prostate cancer cells can evade therapy and gain new phenotypes, namely inhibition of oncoprotein turnover due to elevated activity of ubiquitin-specific peptidases such as USP2.

An emerging body of research positions USP2 as an oncogenic factor across a broad spectrum of tumour types. Mechanistically, a major function of USP2 is to enhance cancer cell proliferation via stabilisation of key substrates involved in cell cycle control, including cyclins A1 and D1, Aurora A, epidermal growth factor receptor and human epidermal growth factor receptor 2 [52, 53, 55, 62–64]. Beyond its pro-growth functions, USP2 antagonises p53 signalling to enhance cancer cell survival by stabilising MDM2 and MDMX [65–68] and, in prostate cancer, it can mitigate oxidative stress induced by chemotherapeutic drugs [69]. USP2 also influences cancer spread by stabilising pro-metastatic factors, including TGFβ receptor, Twist and MMP2 [70–72]. Reflecting its multifaceted oncogenic activities, high expression of USP2 has been linked to poor outcomes in tumours of the upper urothelial tract, stomach and liver [73–75].

Our study provides new insights into the tumour-promoting roles of USP2 and further authenticates it as a therapeutic target. One important finding was that USP2 can promote a glycolytic phenotype in prostate cancer cells, evidenced by elevated expression of glycolysis-related factors at an mRNA and protein level, as well as extracellular flux analysis. USP2 is known to influence systemic control of glucose homeostasis [76, 77], although whether this function relates to its pro-glycolytic activity in prostate cancer remains to be determined. Another discovery arising from our research was that USP2 can influence cell phenotype, including inducing morphological and transcriptional changes that have been linked to epithelial-neuroendocrine transition in prostate cancer. This is reminiscent of earlier reported links between USP2 and cancer cell plasticity. For example, He and colleagues demonstrated that USP2 promotes epithelial-mesenchymal transition (EMT) and stemness in triple-negative breast cancer cells by stabilising the EMT transcription factor Twist, which in turn increases the expression of the stemness factor Bmi1 [70]. More recently, EZH2 was identified as a substrate of USP2 in bladder cancer [78], which is of interest given the prominent role that EZH2 plays in prostate cancer cell plasticity [3]. Thus, we propose that an important oncogenic function of USP2 is to unlock the phenotypic plasticity of cancer cells, a hallmark of malignancy that is closely linked to therapy resistance and immune evasion [7, 79].

Data from patient samples, patient-derived explants, mouse models and cell lines demonstrate that USP2 is negatively regulated by the AR signalling axis. Emerging evidence suggests that genes inhibited by this pathway frequently become important drivers of prostate cancer progression and therapy resistance when they are de-repressed following AR inhibition [3]; key examples of this phenomenon include EZH2, SOX2, BRN2 and FOXA2 [12, 49, 80–83]. Interestingly, an earlier study proposed that USP2 protein levels were increased by androgen treatment in LNCaP cells, although its regulation at the mRNA level in this model was not evaluated [84]. The reasons for the apparent discordance between our findings and this previous work are unclear. Notably, Graner and colleagues used an in-house antibody to evaluate USP2 protein that is no longer available (M. Loda, personal communication) and the commercial antibodies we tested were unable to reliably and/or specifically detect USP2 (data not shown); as a result, we were unable to determine whether androgen treatment caused differential effects on USP2 mRNA versus protein. Notwithstanding this issue, our experimental analyses and mining of contemporary clinical datasets provide strong evidence for an inverse relationship between USP2 expression and AR signalling. Since androgen-mediated regulation of USP2 would influence approaches to therapeutically target this factor in prostate cancer, elucidating the mechanism(s) underlying this relationship is an important area of future research.

Numerous USP2 inhibitors have been reported, but ML364 is the best characterised to date [11]. ML364, which was identified in a high-throughput screen, has low micromolar activity against USP2 in vitro but no detectable activity against >100 kinases [52]. The promising pharmacological properties of ML364 have prompted its evaluation in multiple cancer types; indeed, it has been reported to elicit anti-growth and anti-metastasis effects in in vivo pre-clinical models of breast, colorectal, lung and liver cancer and mantle cell lymphoma [52, 64, 70, 71, 85, 86]. Our study extends this earlier work by demonstrating that ML364 inhibits the growth of an aggressive, AR-null model of prostate cancer without causing any noticeable toxicity. Thus, further evaluation of this small molecule is warranted.

In summary, we identify USP2 as an androgen-repressed ubiquitin-specific peptidase that is strongly associated with aggressive, AR-independent subtypes of prostate cancer. The pleiotropic oncogenic functions of USP2, mediated via its suite of cancer-relevant substrates, make it an attractive target for future investigation and therapeutic development.

## Supplementary information

Supplementary Tables S1-S5

Supplementary Figures S1-S5

Supplementary Figure S3 Original Data

## Data Availability

RNA-seq data generated for this study are available from NCBI’s Gene Expression Omnibus (GSE297844).
