# Supplementary material for: USP2 is an androgen-repressed survival factor that stabilises oncoproteins to facilitate therapy resistance in prostate cancer
Source: Cell Death Dis. 2026 Jun 2;17(1):687. doi: 10.1038/s41419-026-08923-7 (PMC13443806; doi:10.1038/s41419-026-08923-7)
Supplement: Supplementary file 1 [file 41419_2026_8923_MOESM2_ESM.pdf]

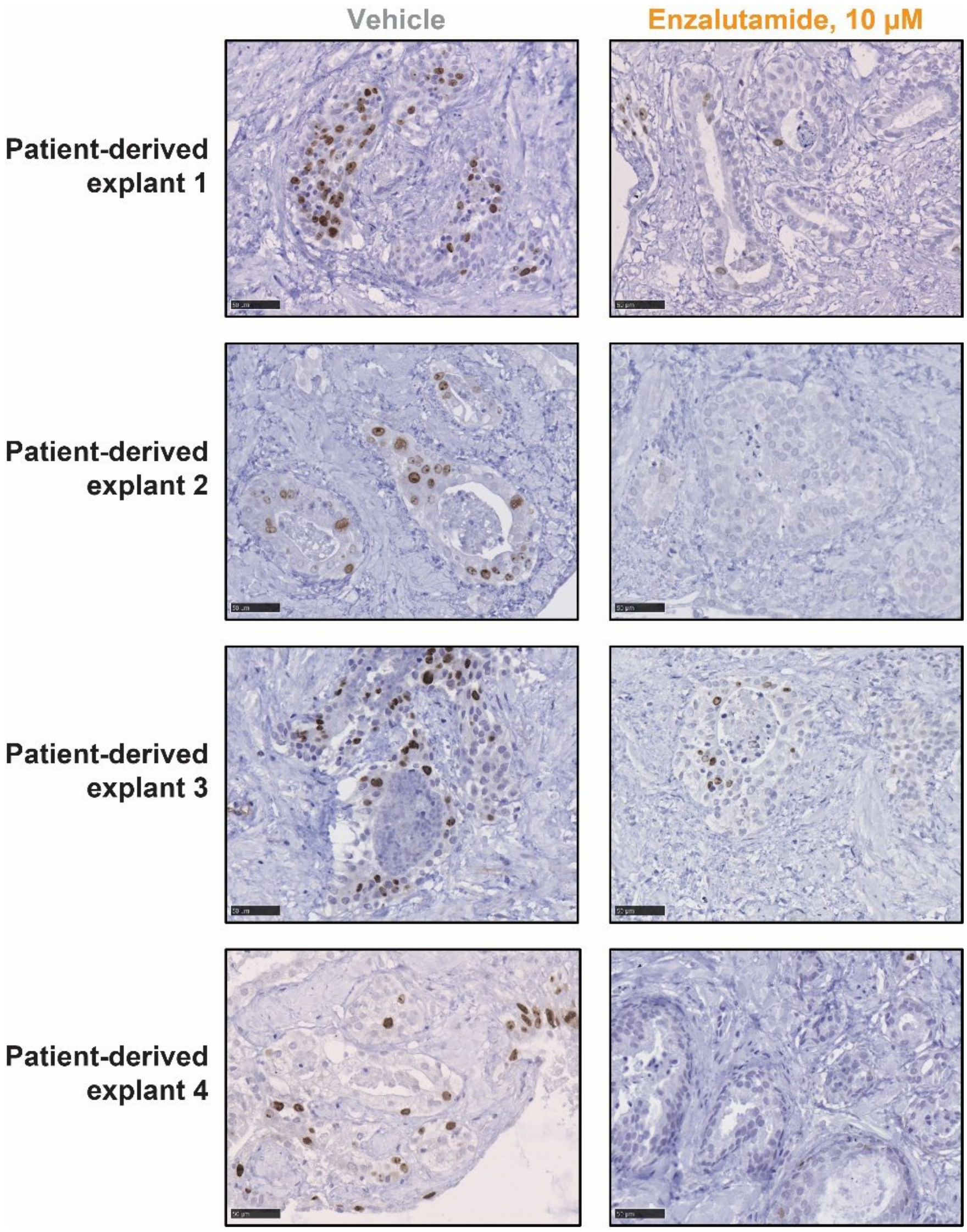

Figure S1. Reduction of Ki67 protein in response to enzalutamide in patient-derived explants (PDEs). Ki67 levels in tumours from 12 patients were measured by immunohistochemistry (IHC). Representative IHC images from 4 of the PDEs are shown (scale bars represent 50 µm).

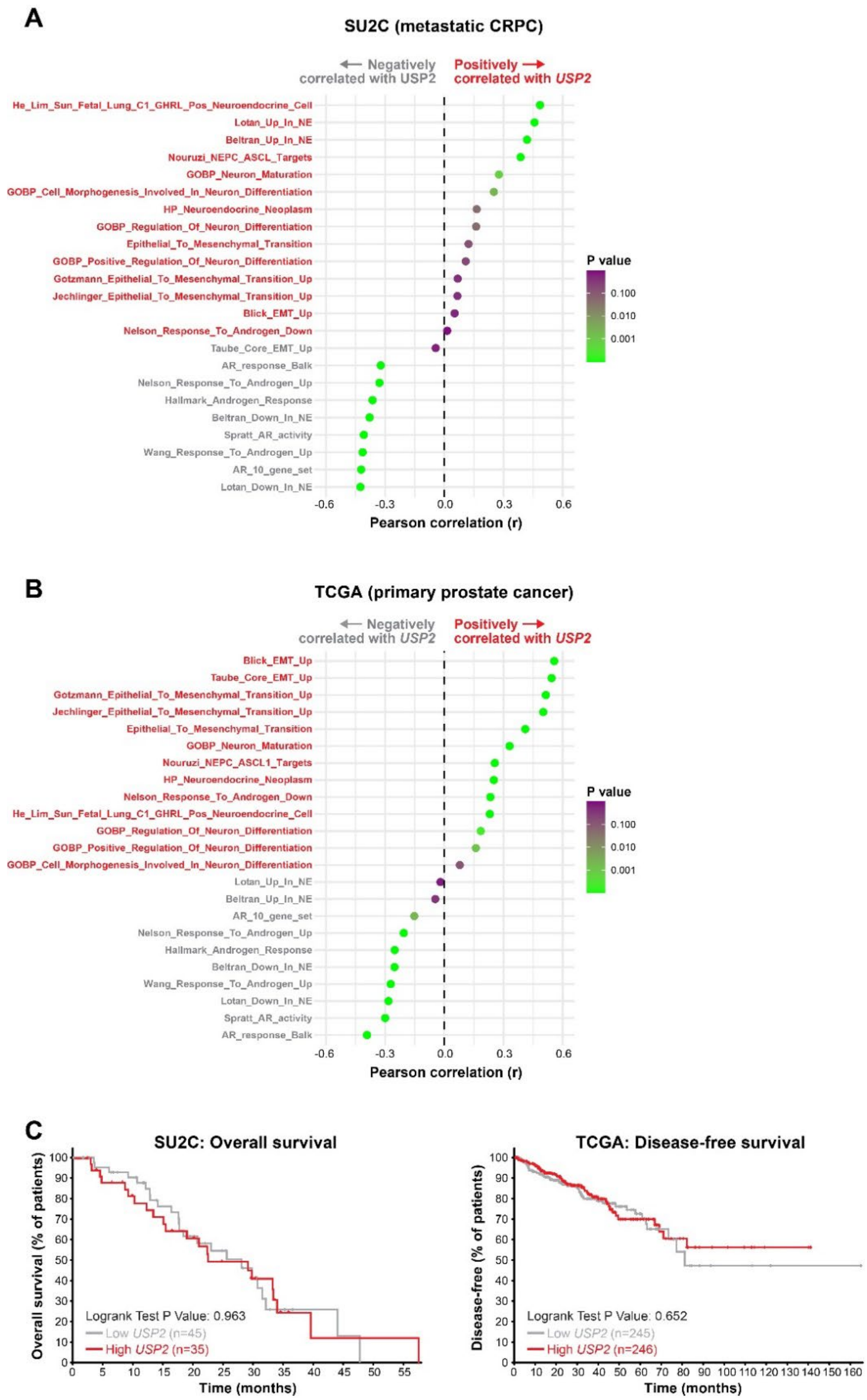

Figure S2. (A-B) *USP2* expression was positive correlated with neuroendocrine prostate cancer (NEPC) and epithelial-mesenchymal transition (EMT) and inversely correlated with AR activity in primary tumours in (A) metastatic CRPC (Stand Up 2 Cancer (SU2C) cohort; reference #24) and (B) primary prostate cancer (The Cancer Genome Atlas (TCGA) cohort; reference #23). Pearson correlation coefficient (r) values represent associations between *USP2* mRNA levels and Single Sample GSEA scores for the indicated gene sets. A curated selection of gene sets associated with NEPC, EMT, neuronal/neuroendocrine differentiation, stem-ness and androgen receptor (AR) activity was used. **(C)** *USP2* mRNA levels were not associated with overall survival (SU2C) or disease-free survival (TCGA). Survival analyses were done using cBioPortal (reference #22). Groups (low *USP2*, high *USP2*) were stratified using median levels of *USP2*; note that this stratification was done prior to removing individuals without outcome data, resulting in different group sizes in the SU2C cohort.

Figure S3. Uncropped immunoblots. See accompanying file.

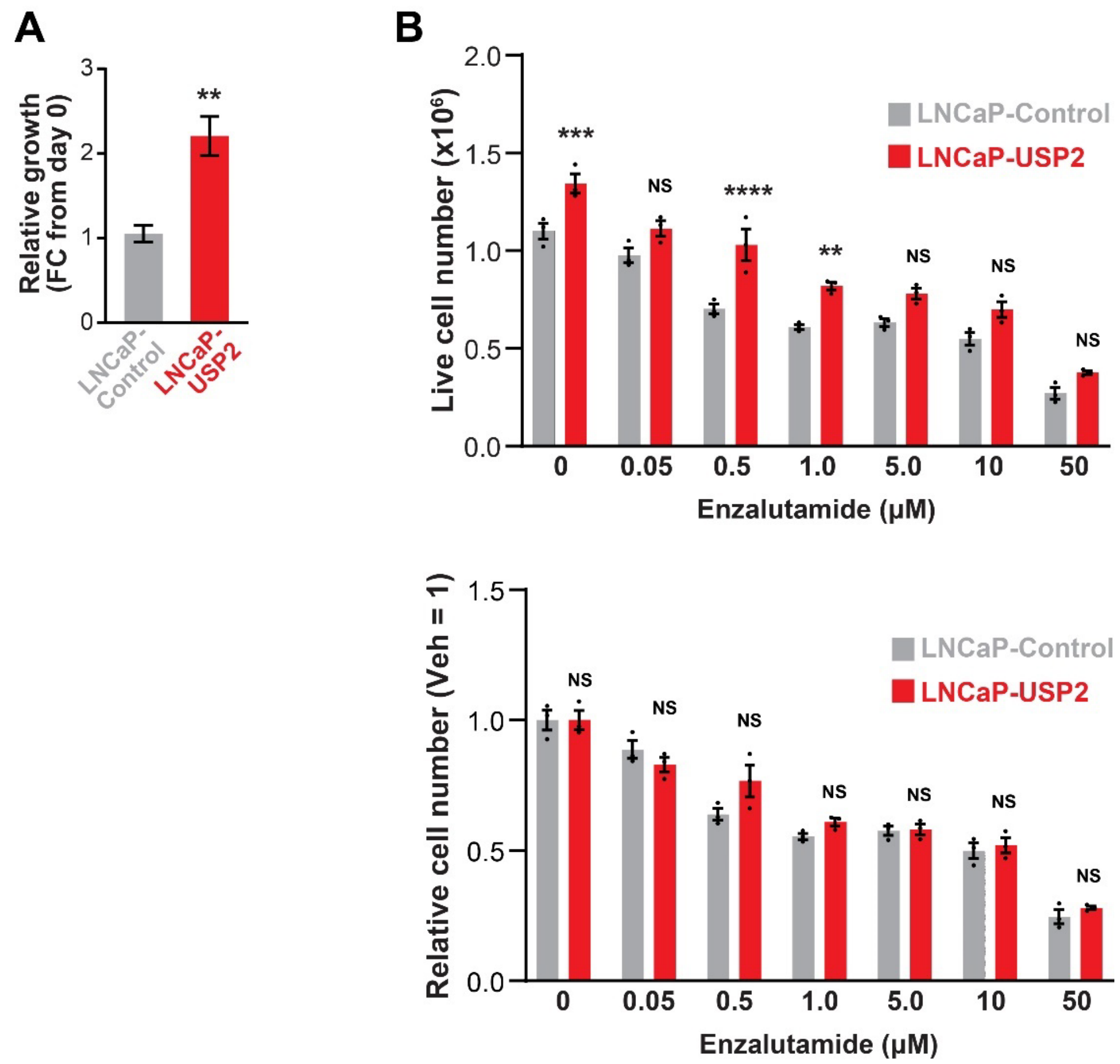

Figure S4. (A) Relative growth of LNCaP-Control and LNCaP-USP2 cells in charcoal-stripped (androgen deprived) media. Fold growth over a period of 4 days was calculated relative to the number of cells present at day 0. Data were analysed using an unpaired t test. **(B)** Cell number in LNCaP-Control and LNCaP-USP2 cells after 3 days of treatment with the indicated doses of enzalutamide (upper graph). Cell number was determined by trypan blue assay. Two-way ANOVA and Sidak's multiple comparison tests were used to compare LNCaP-USP2 relative to LNCaP-Control. The lower graph shows data from the upper graph but corrected to growth in vehicle (basal conditions); after this correction, USP2 overexpression was not associated with a growth advantage in the presence of enzalutamide. In all panels: **, $p < 0.01$; ***, $p < 0.001$; ****, $p < 0.0001$; NS, not significant.

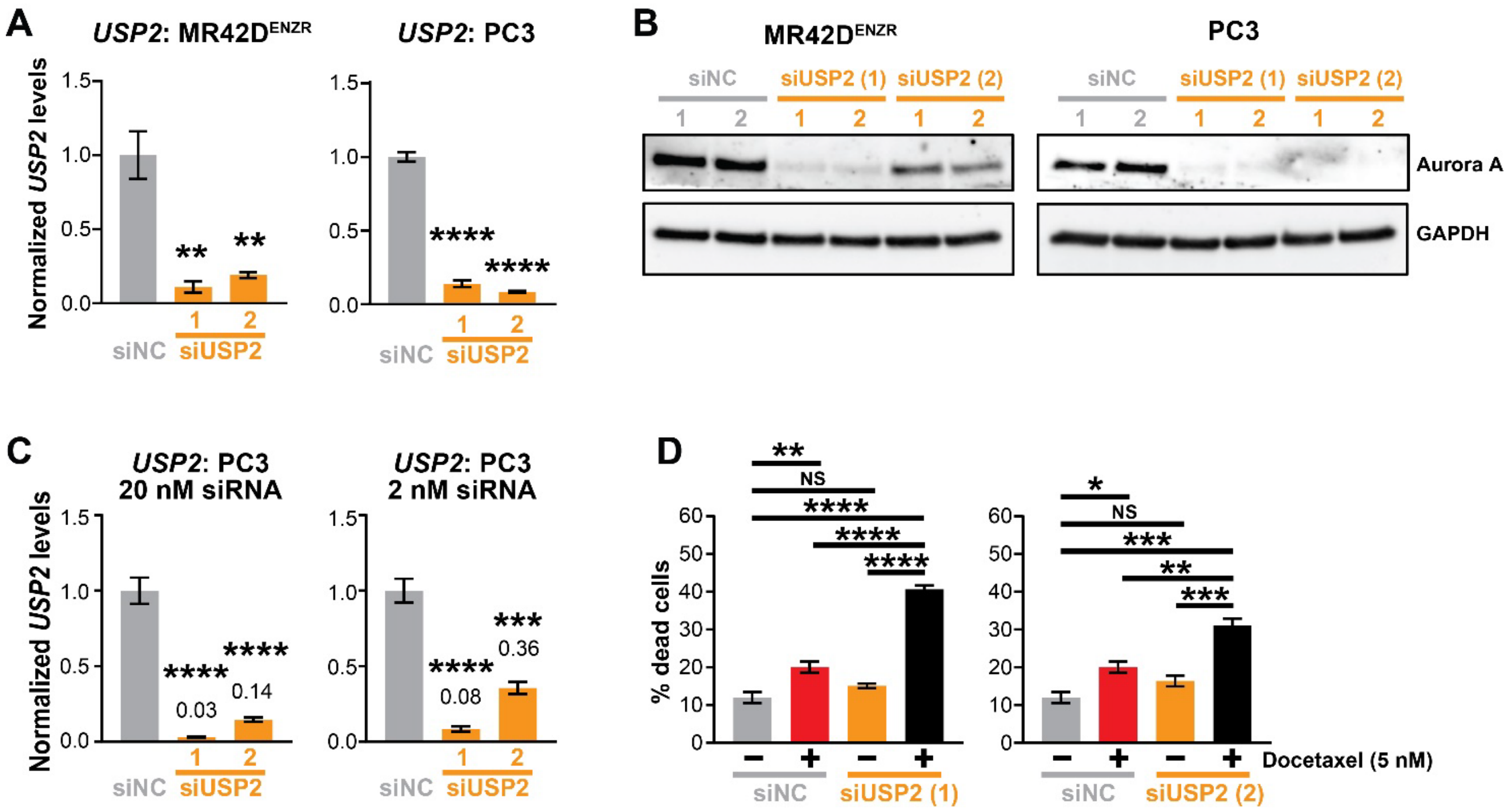

Figure S5. (A) Quantitative RT-PCR showing reduction in *USP2* mRNA levels in MR42D^{ENZR} and PC3 cells transfected for 3 days with 20 nM of two distinct siRNAs targeting USP2 (siUSP2 1 and 2). Data were normalised to *GAPDH*. Error bars are ± standard error of the mean (SEM). Groups were compared using ANOVA and Dunnett's multiple comparison tests. **(B)** Immunoblotting showing loss of Aurora A in MR42D^{ENZR} and PC3 cells transfected for 3 days with 20 nM of two distinct siRNAs targeting USP2. For each siRNA, 2 biological replicates are shown. GAPDH was used as a loading control. **(C)** Quantitative RT-PCR showing reduction in *USP2* mRNA levels in PC3 cells transfected for 3 days with two different doses (20 nM and 2 nM) of siRNAs targeting USP2. Data were normalised to *GAPDH*. Error bars are ± standard error of the mean (SEM). Groups were compared using ANOVA and Dunnett's multiple comparison tests. Numbers on top of bars indicate values relative to siNC (set to 1). **(D)** Knockdown of USP2 sensitises PC3 cells to docetaxel. Cells were transfected with 2 nM of the indicated siRNAs for 3 days, after which they were treated with docetaxel for an additional 3 days prior to evaluating cell death by trypan blue assay. Error bars represent ±SEM and the data are representative of three independent experiments. Groups were compared using ANOVA and Tukey's multiple comparison tests. In all panels: *, $p < 0.05$; **, $p < 0.01$; ***, $p < 0.001$; ****, $p < 0.0001$; NS, not significant.
