# Supplementary material for: USP2 is an androgen-repressed survival factor that stabilises oncoproteins to facilitate therapy resistance in prostate cancer
Source: Cell Death Dis. 2026 Jun 2;17(1):687. doi: 10.1038/s41419-026-08923-7 (PMC13443806; doi:10.1038/s41419-026-08923-7)
Supplement: Supplementary file 2 [file 41419_2026_8923_MOESM3_ESM.pdf]

Figure S3. Uncropped immunoblots.

From Figure 3A

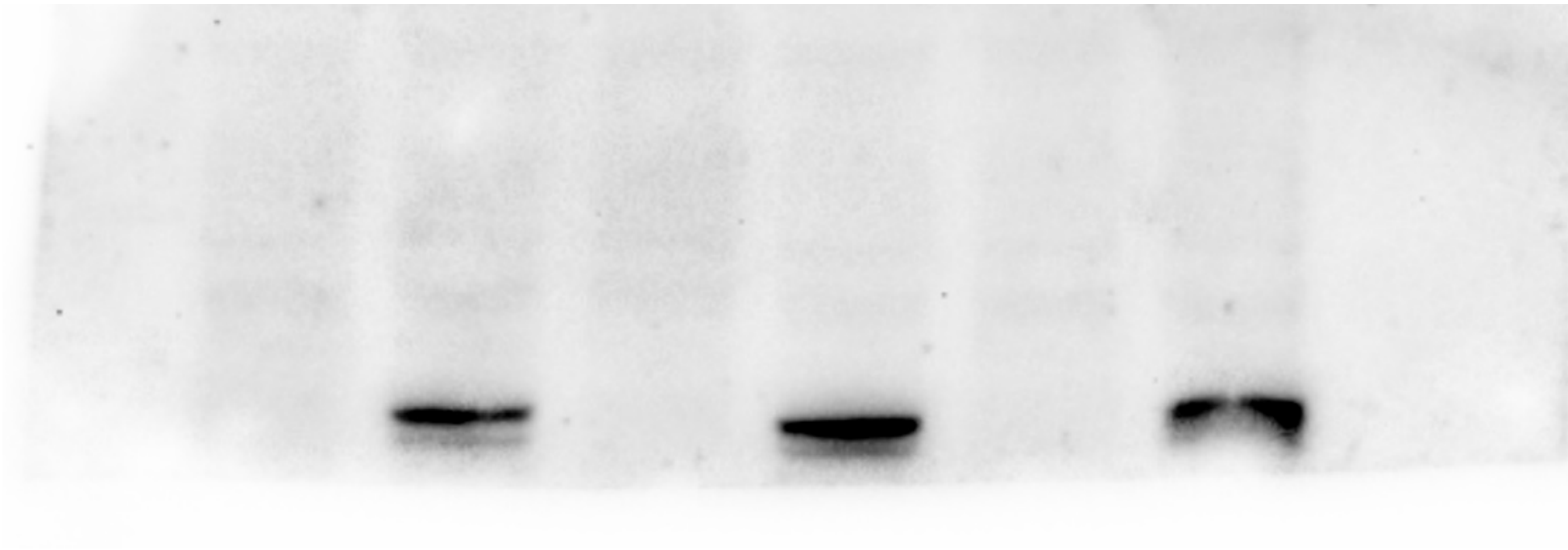

USP2 (Flag): LNCaP-control & LNCaP-USP2

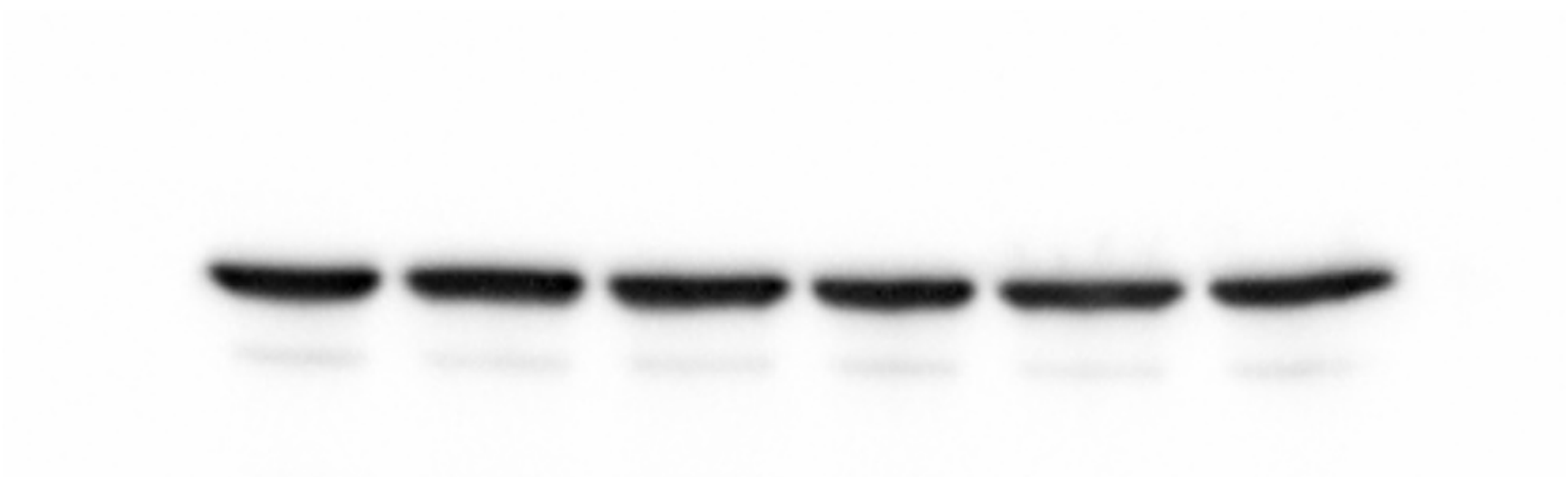

Tubulin: LNCaP-control & LNCaP-USP2

From Figure 4A

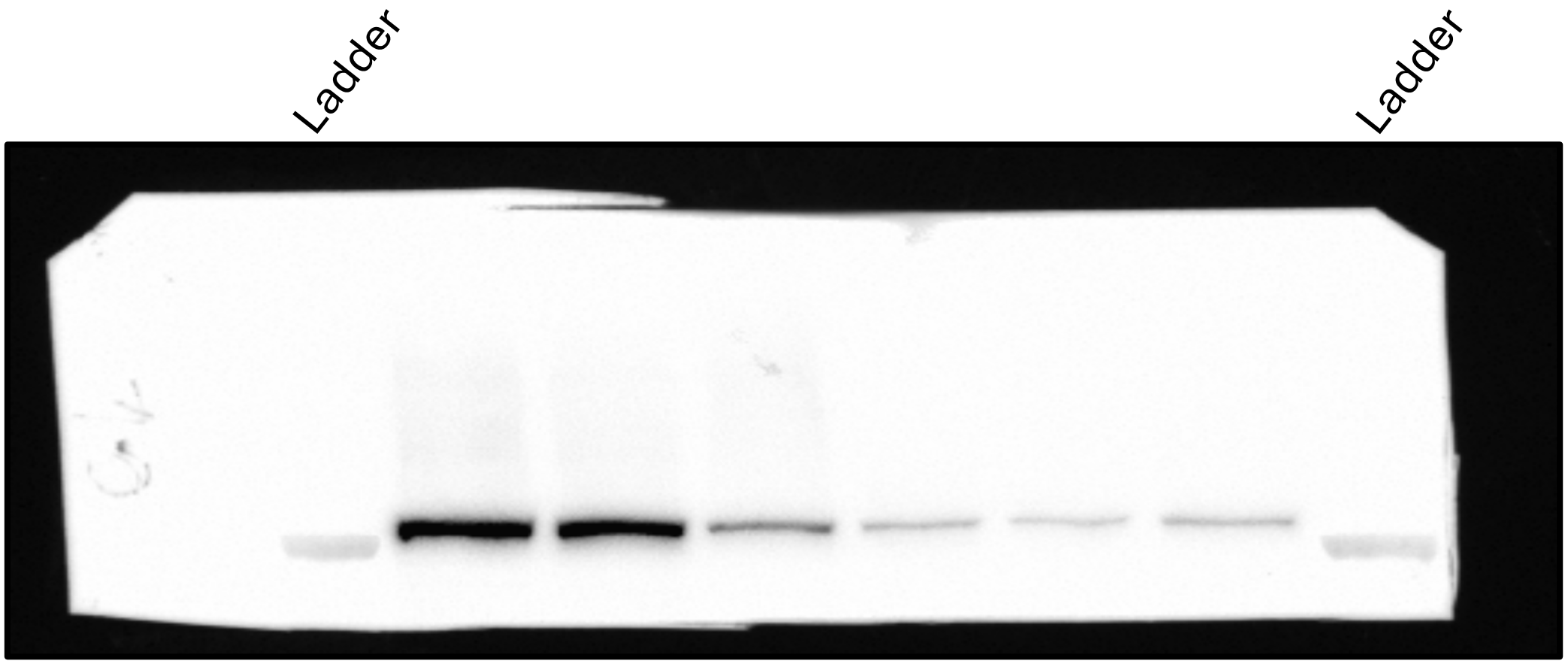

FAS: MR42D 72 h

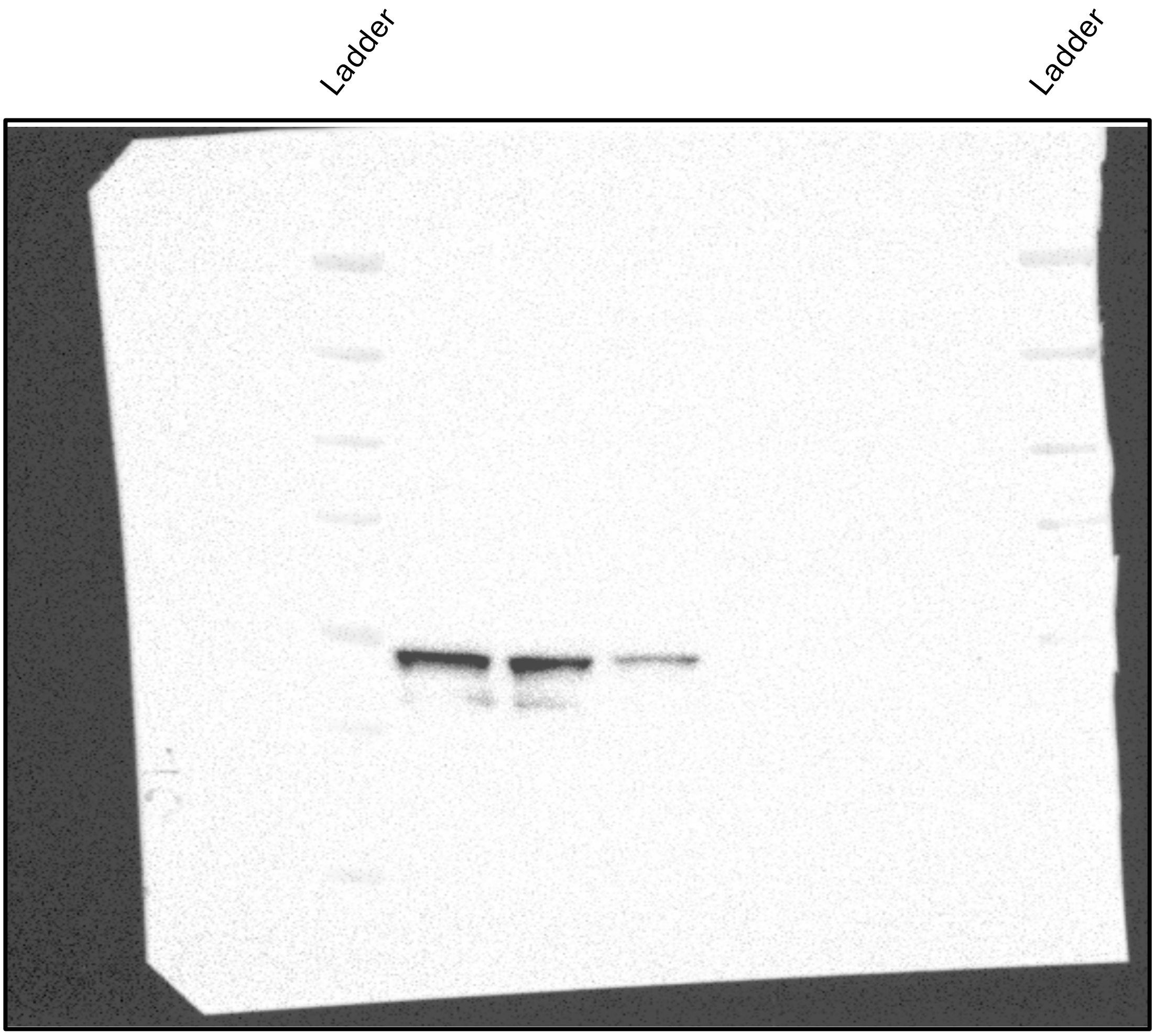

Aurora A: MR42D 72 h

From Figure 4A cont.

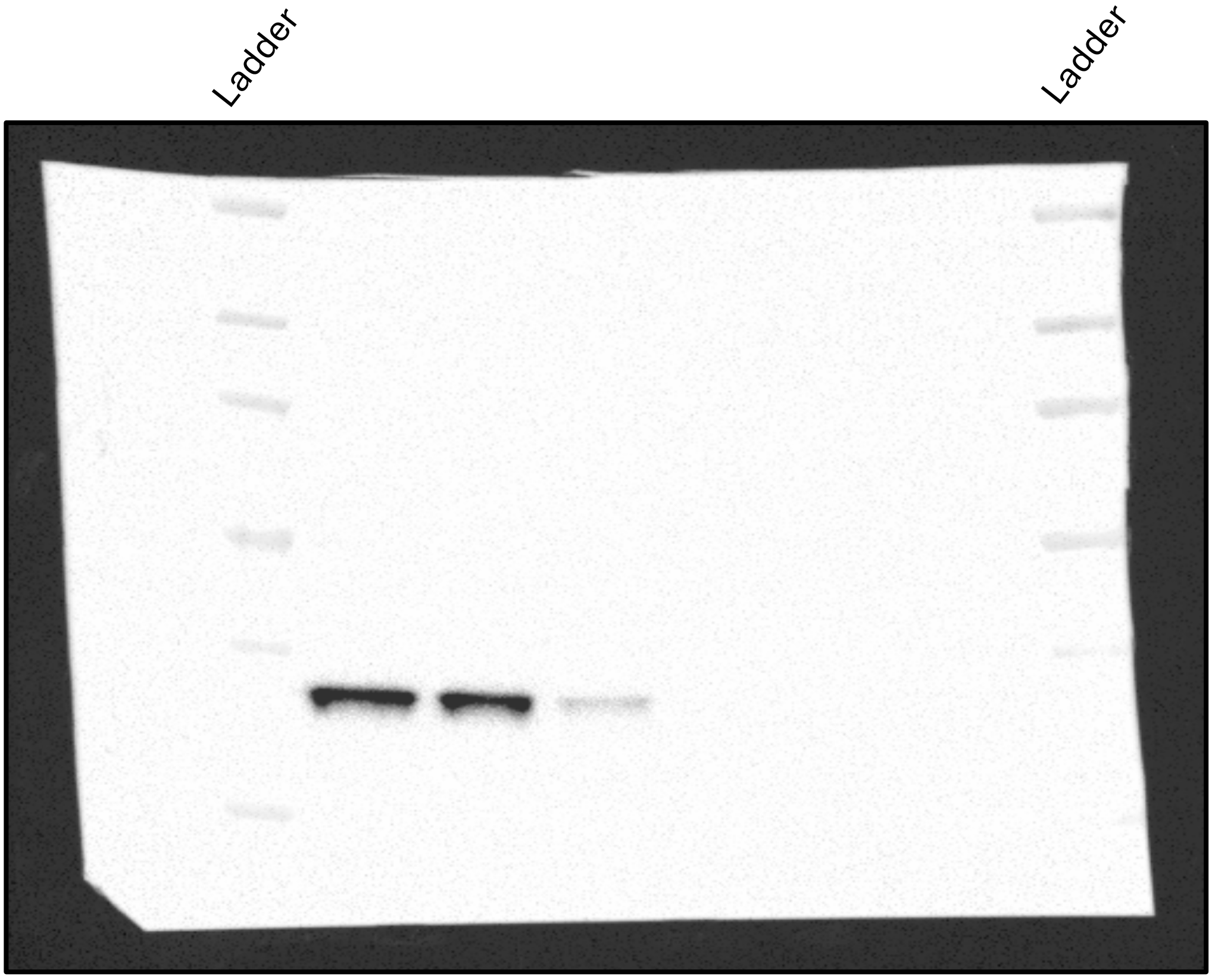

Cyclin D1: MR42D 72 h

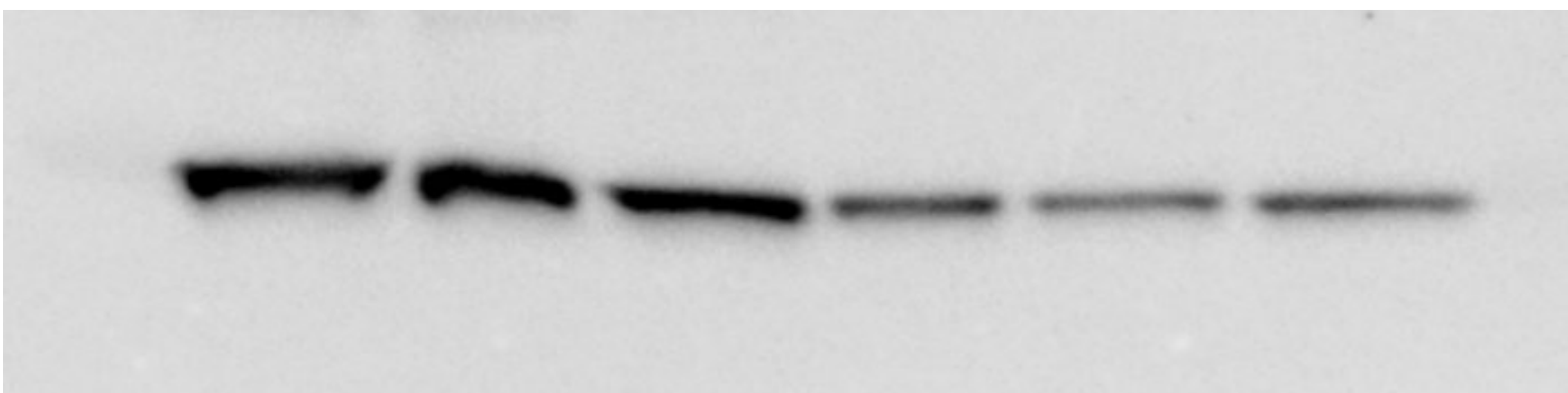

GAPDH/MR42D 72 h

From Figure 4A cont.

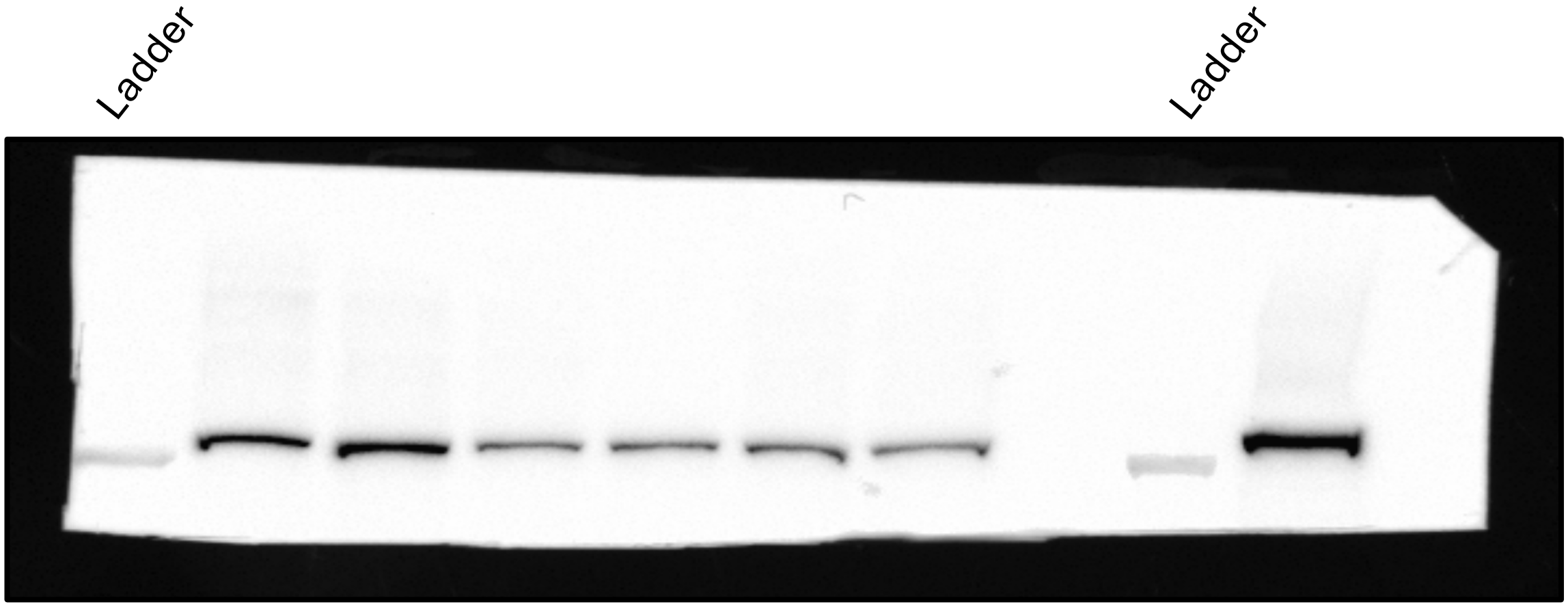

FAS: PC3 72 h

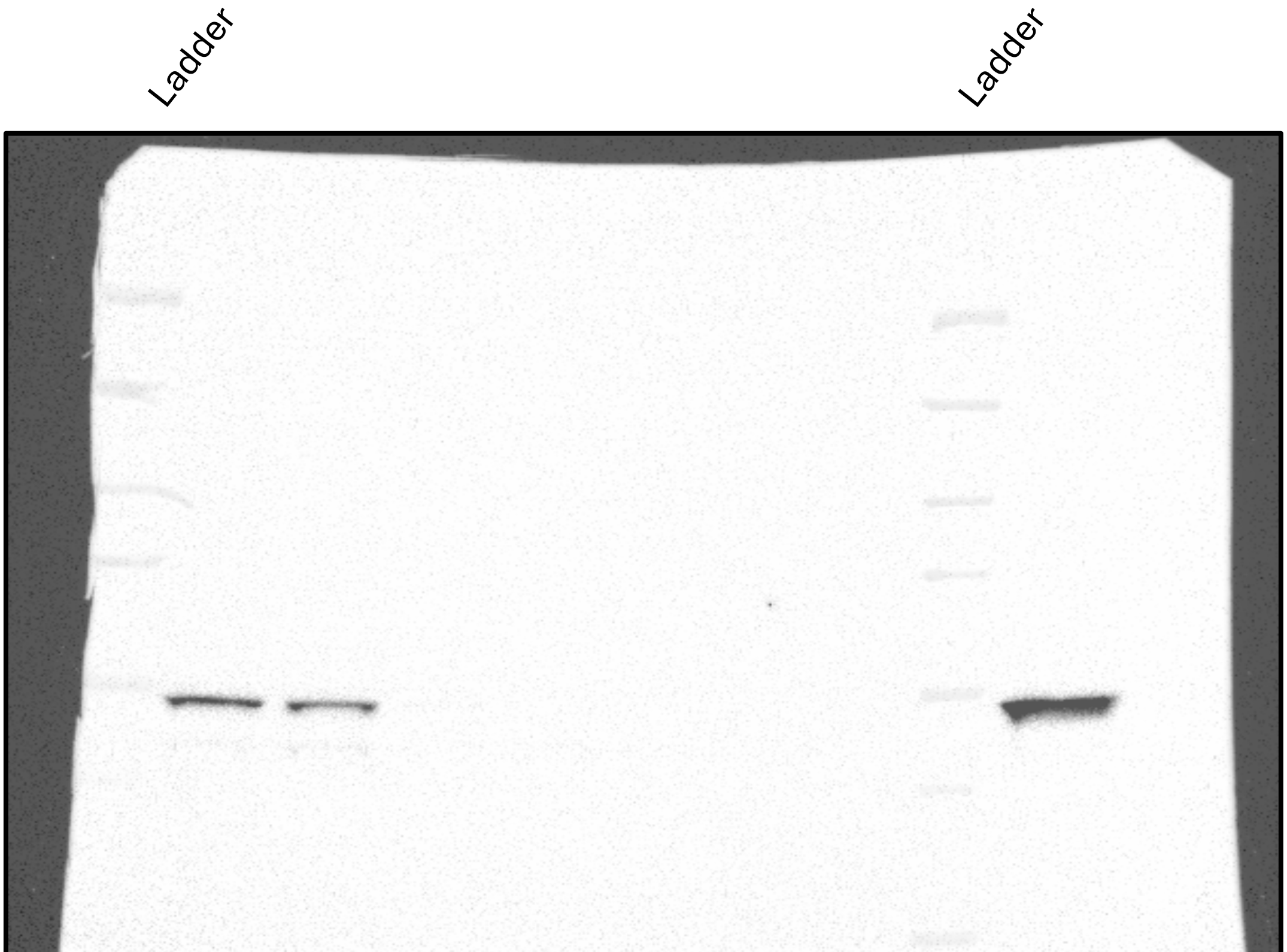

Aurora A: PC3 72 h

From Figure 4A cont.

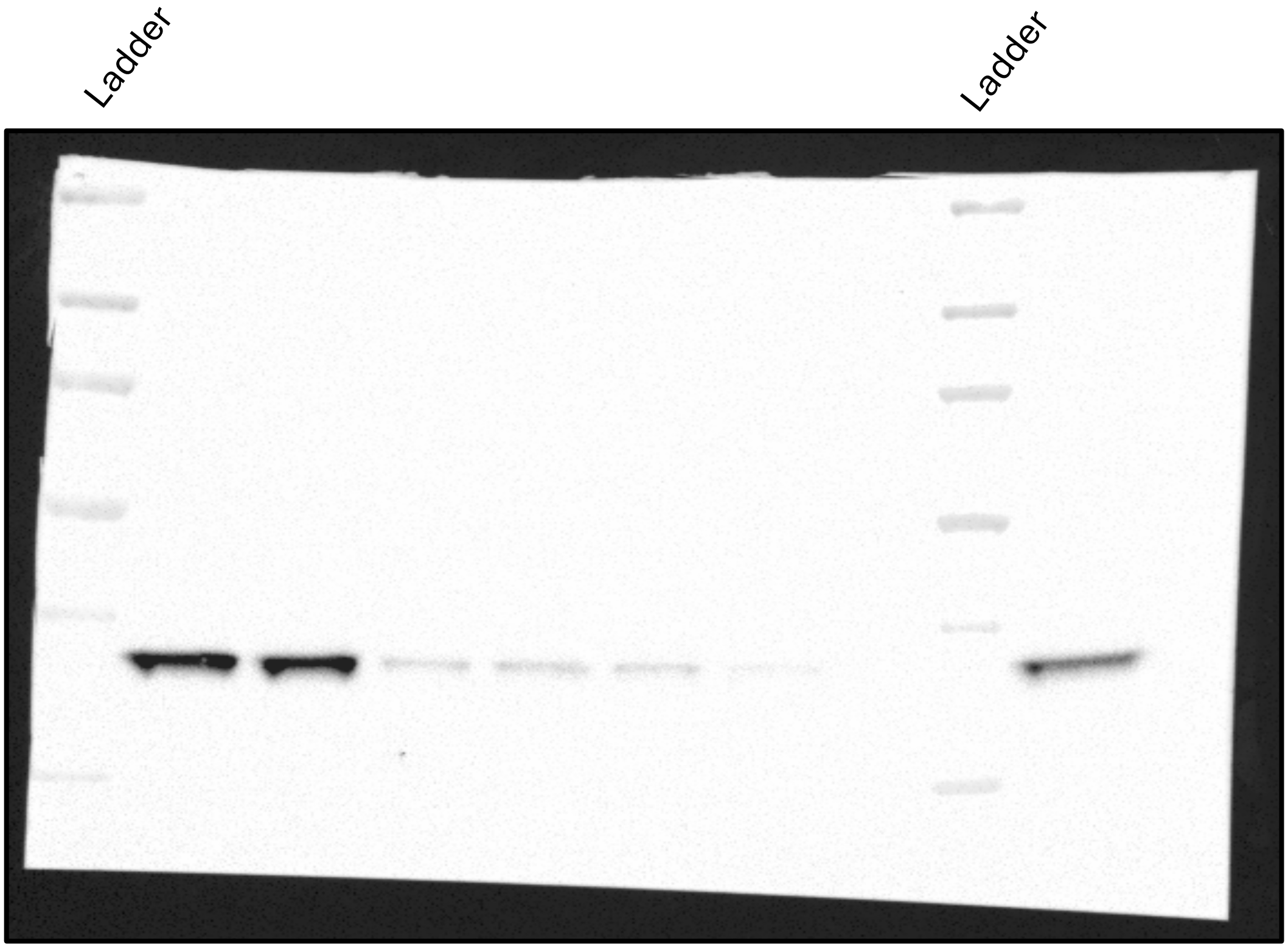

Cyclin D1: PC3 72 h

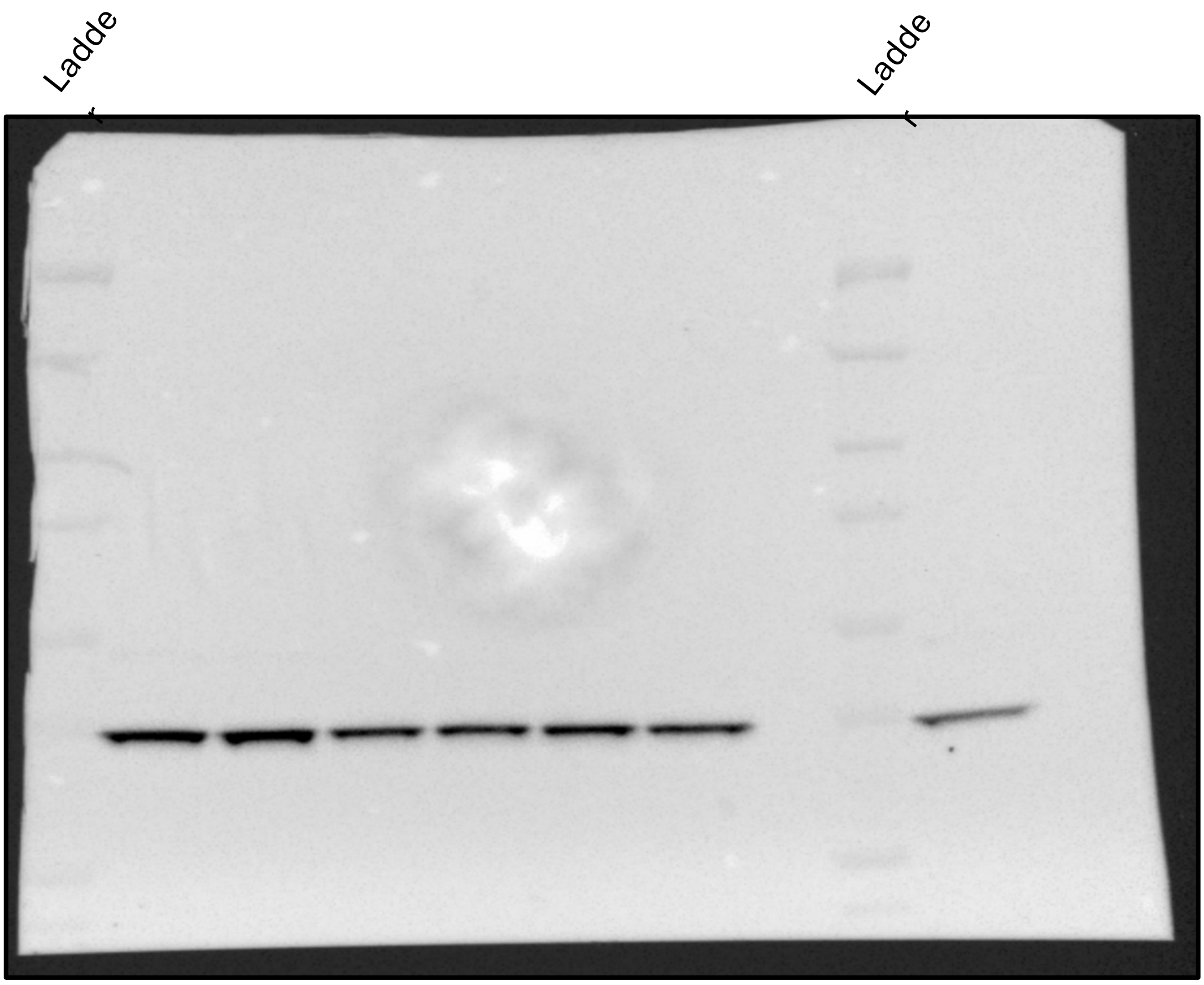

GAPDH: PC3 72 h

From Figure 4B

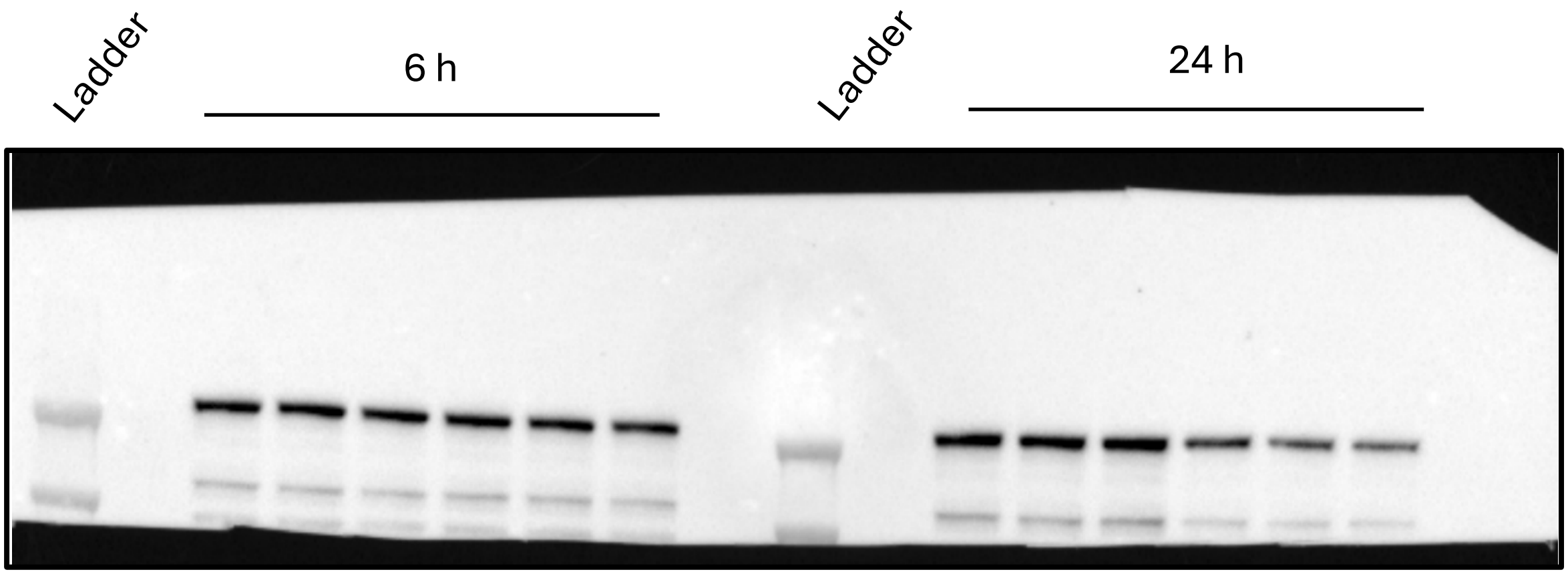

FAS: MR42D 24 h

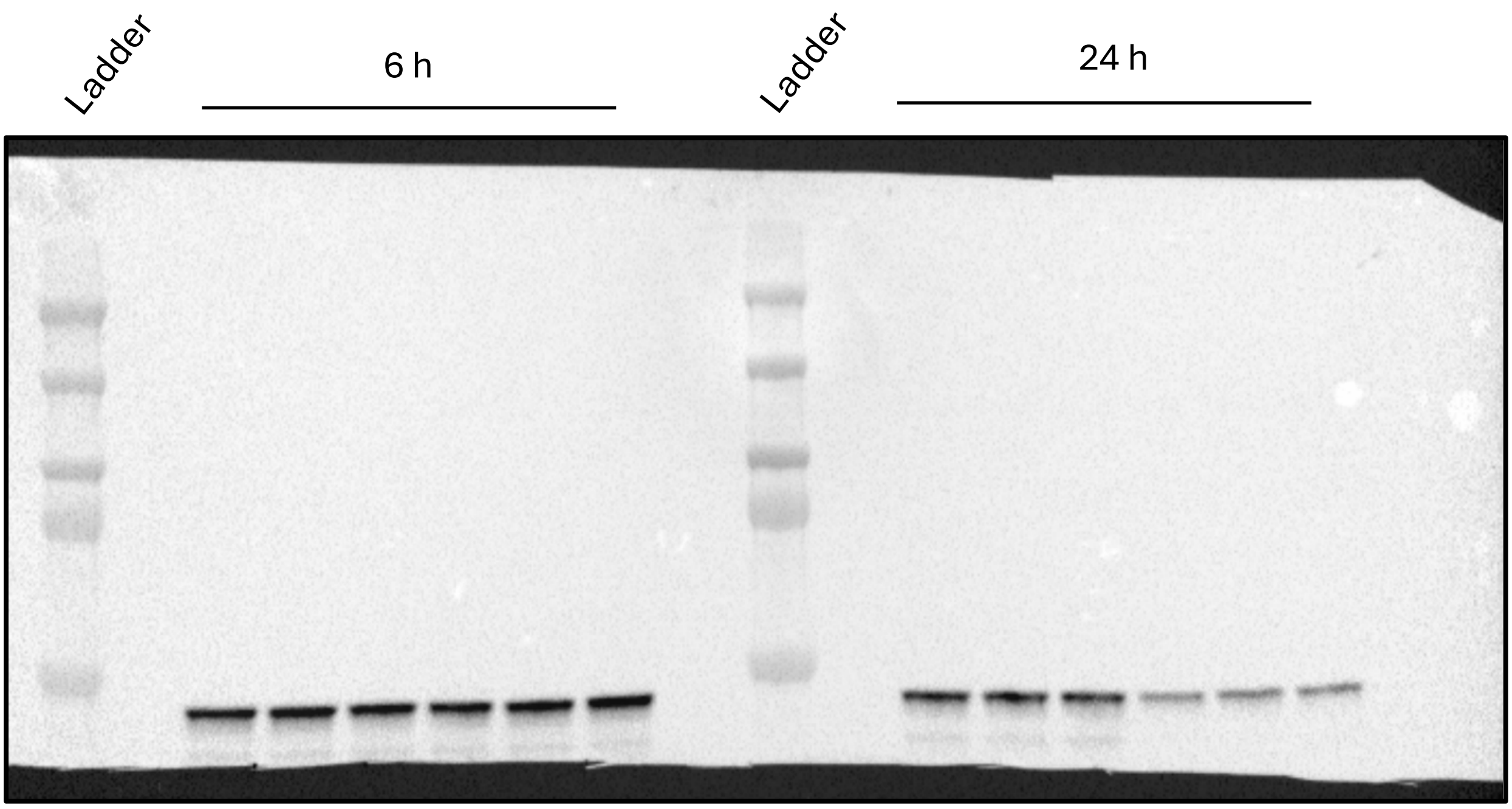

Aurora A: MR42D 24 h

From Figure 4B cont.

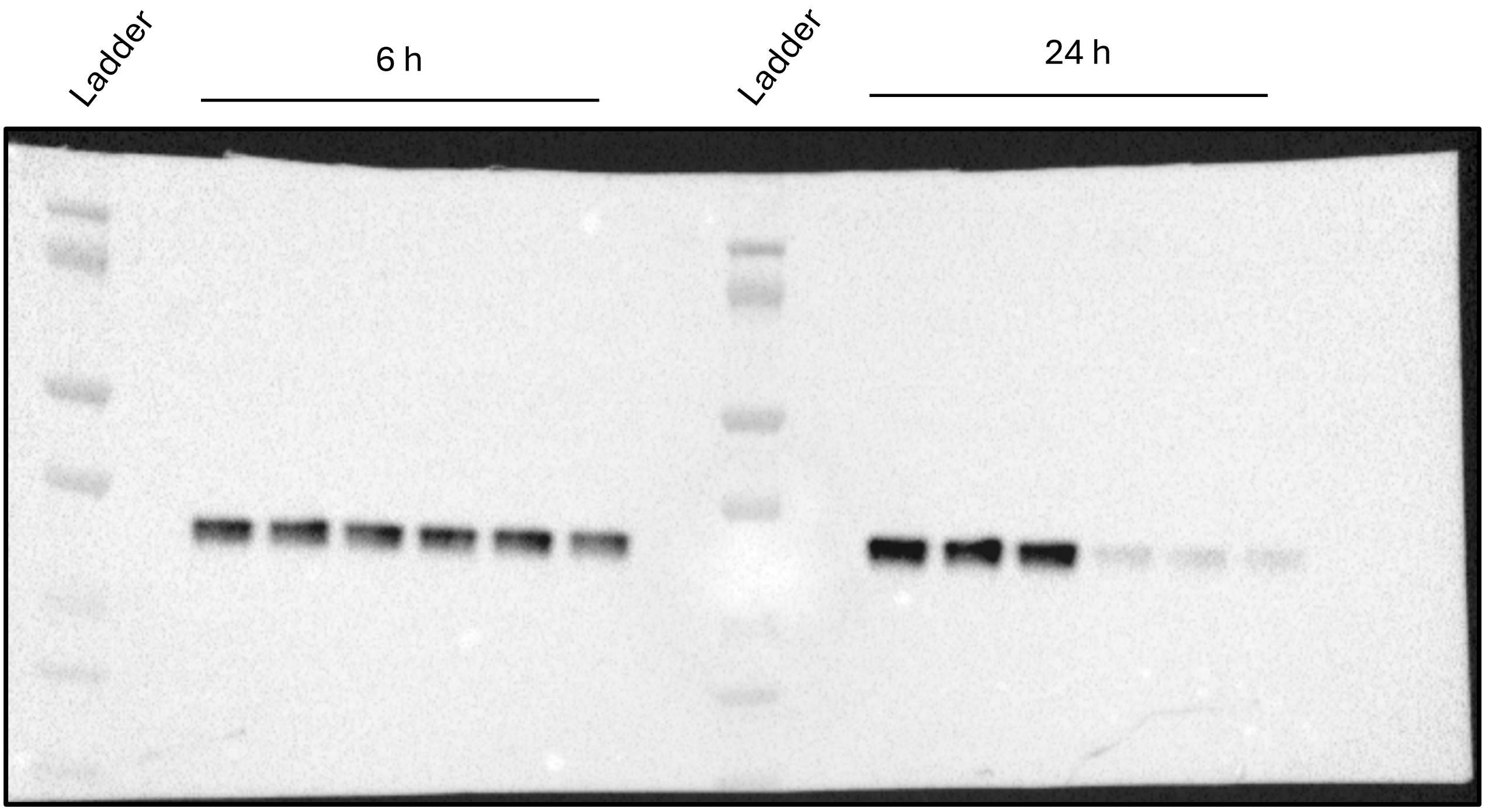

Cyclin D1: MR42D 24 h

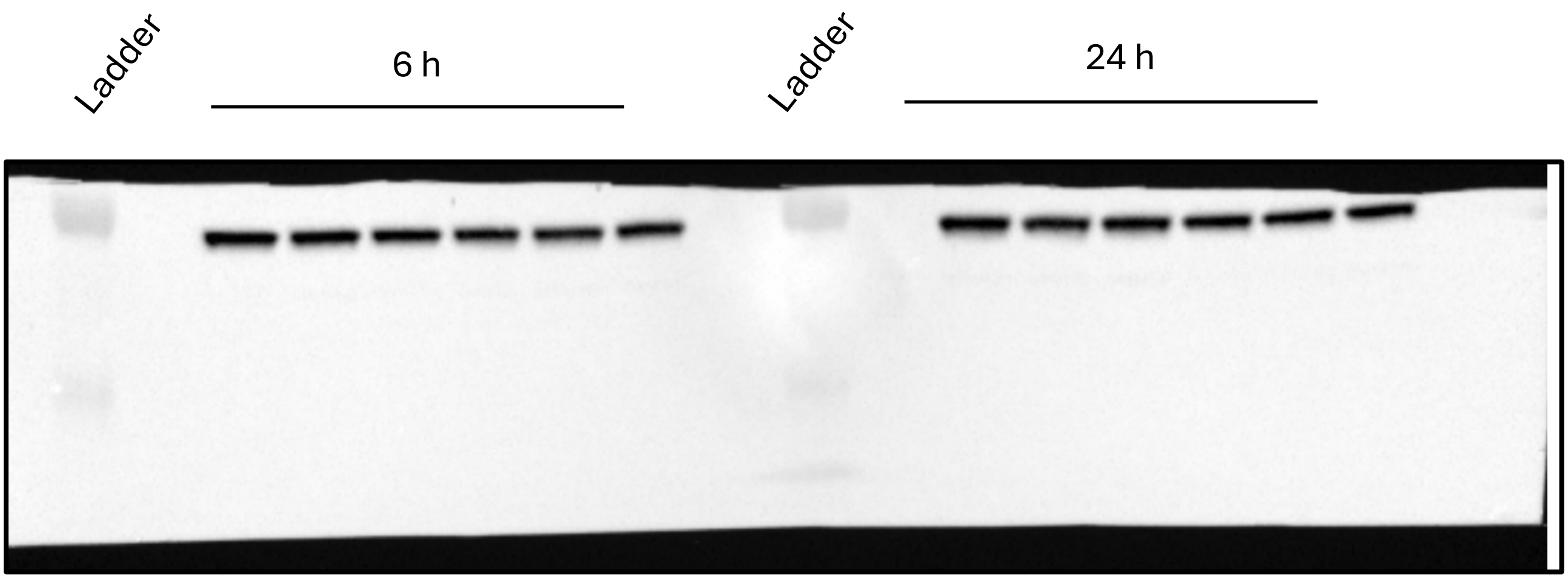

GAPDH: MR42D 24 h

From Figure 4G

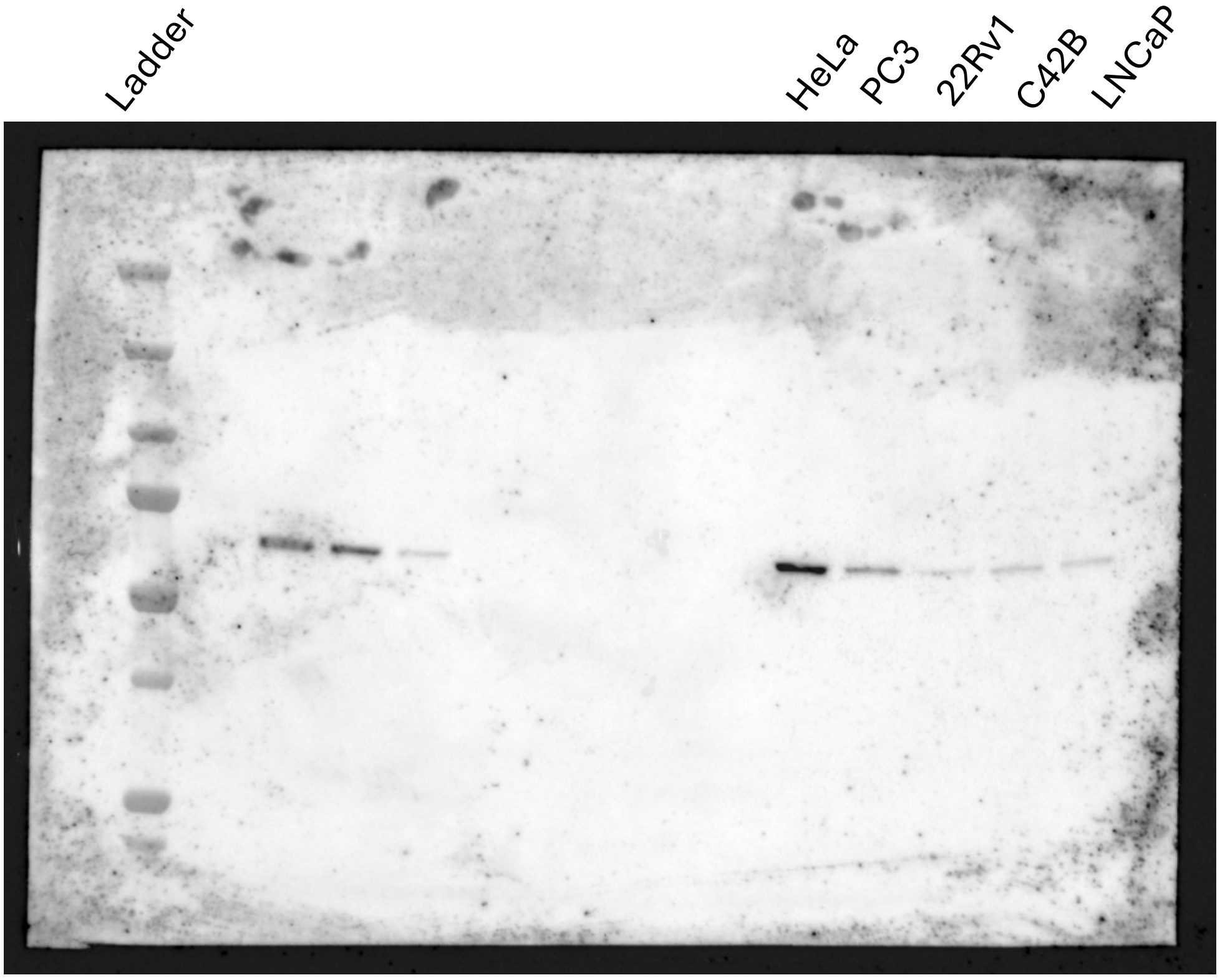

PLK-1: MR42D

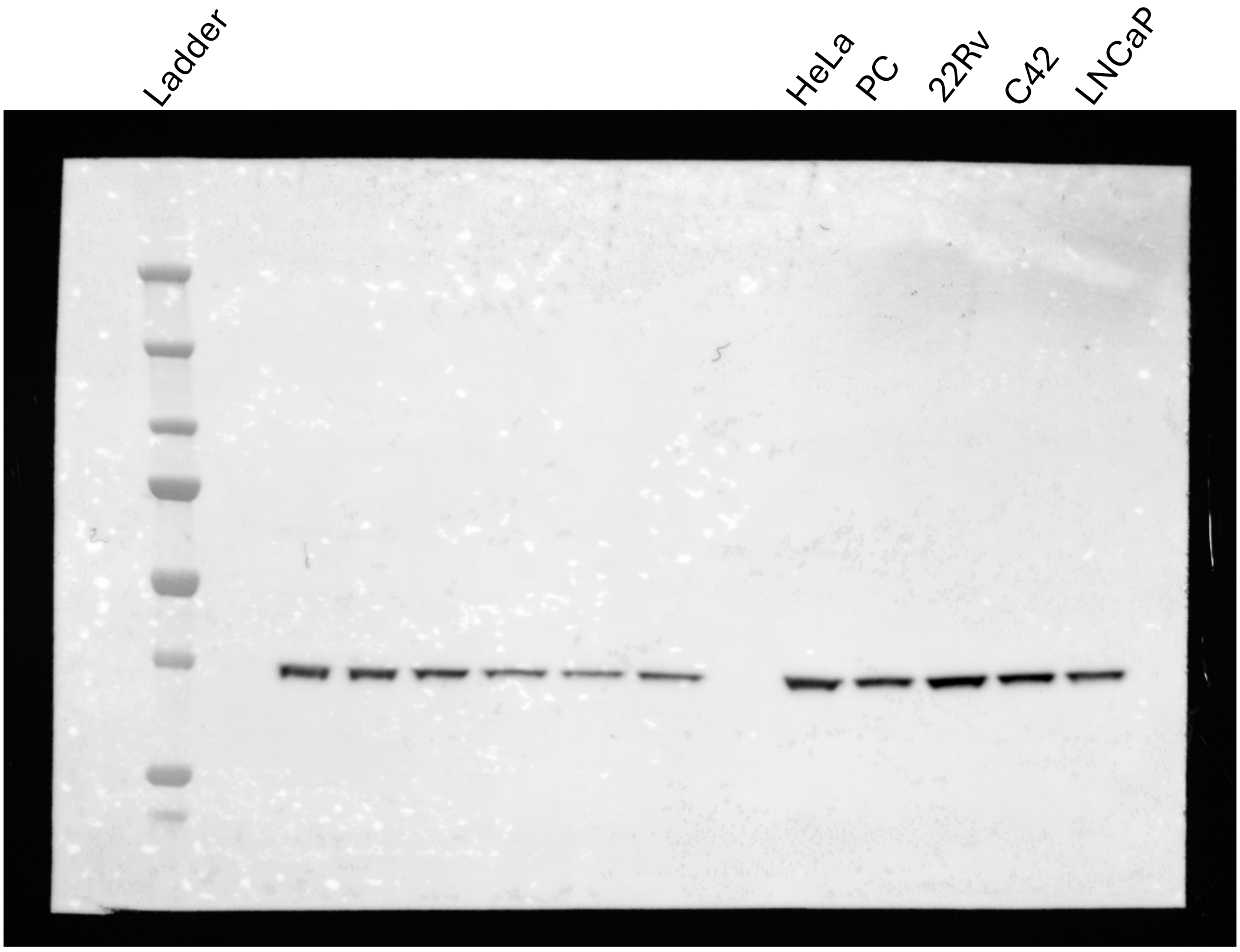

GAPDH: MR42D

From Figure 4G cont.

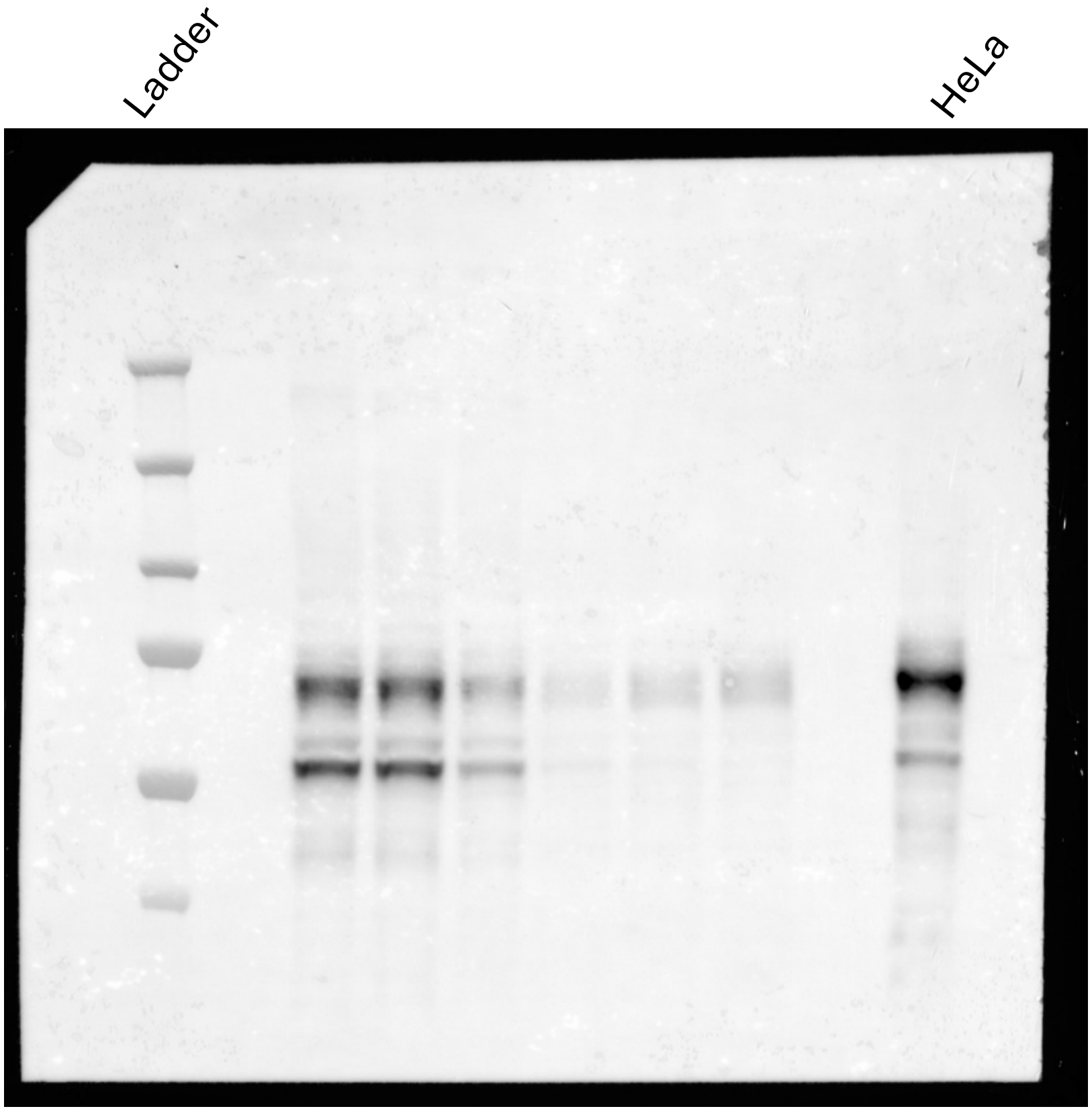

PGRN: MR42D

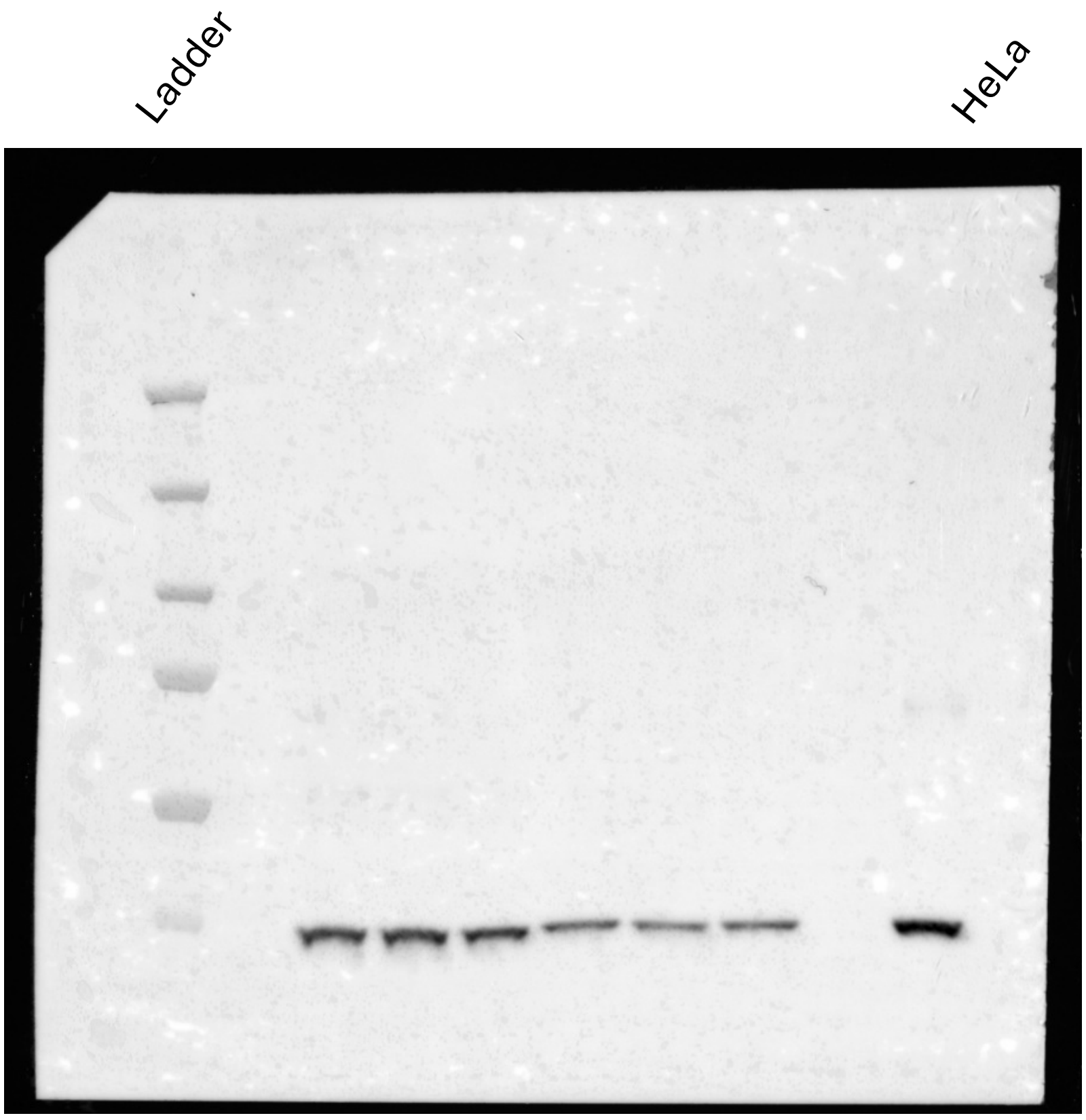

GAPDH: MR42D

From Figure 5K

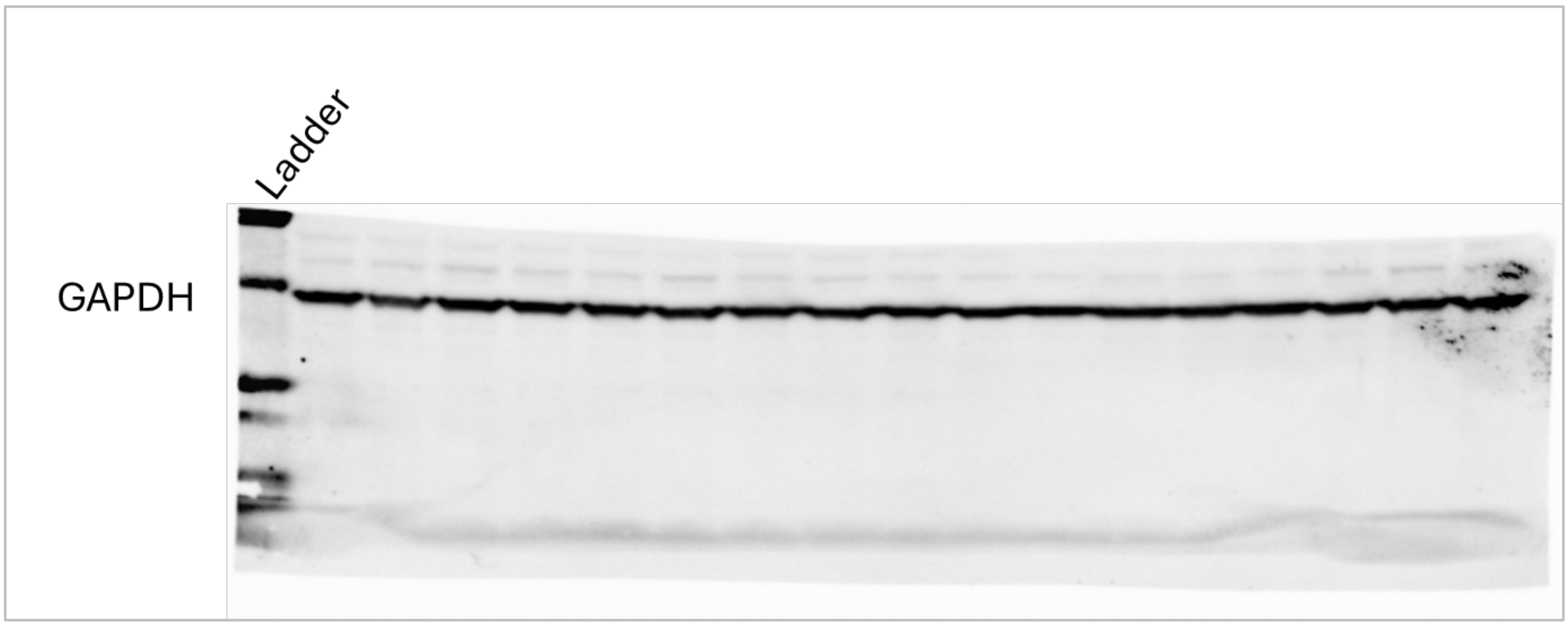

GAPDH: PC3 xenografts

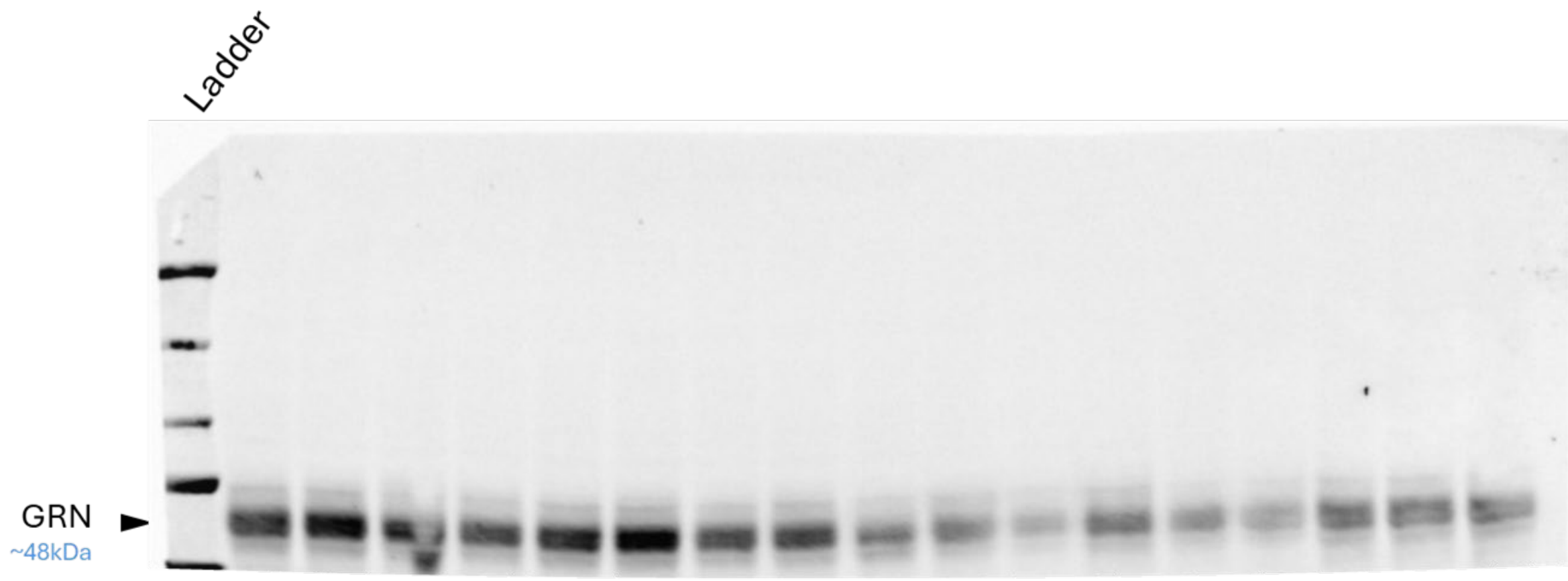

GRN: PC3 xenografts

From Figure 5K cont.

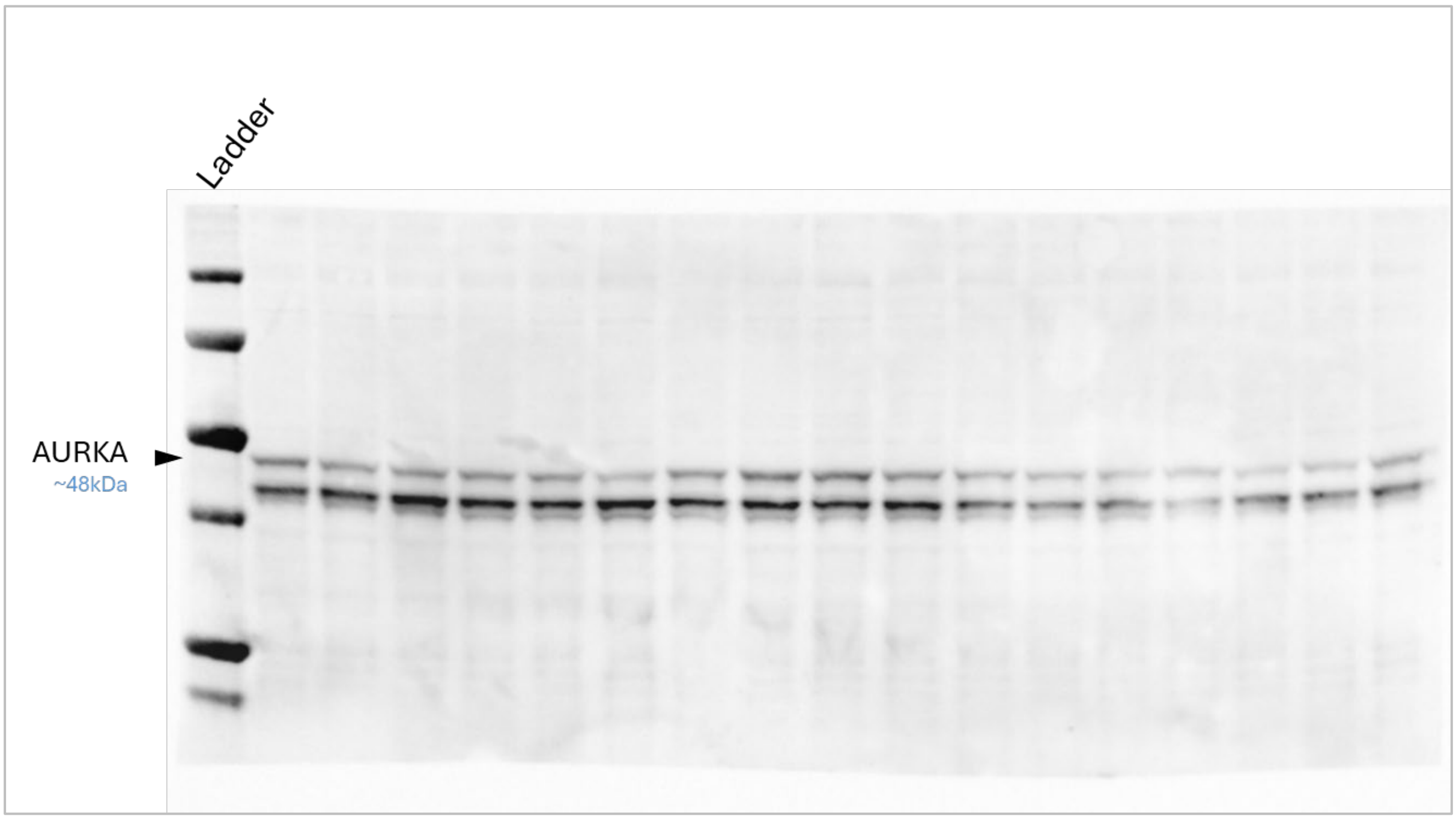

Aurora A: PC3 xenografts

From Figure S5B

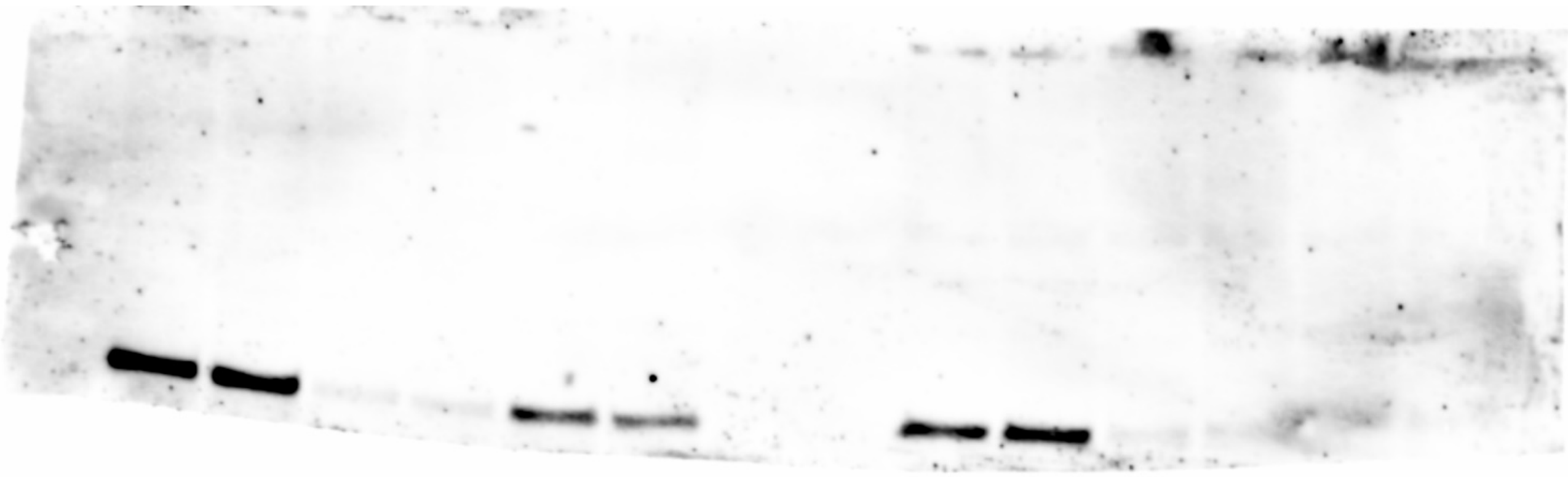

Aurora A/MR42D Aurora A/PC3

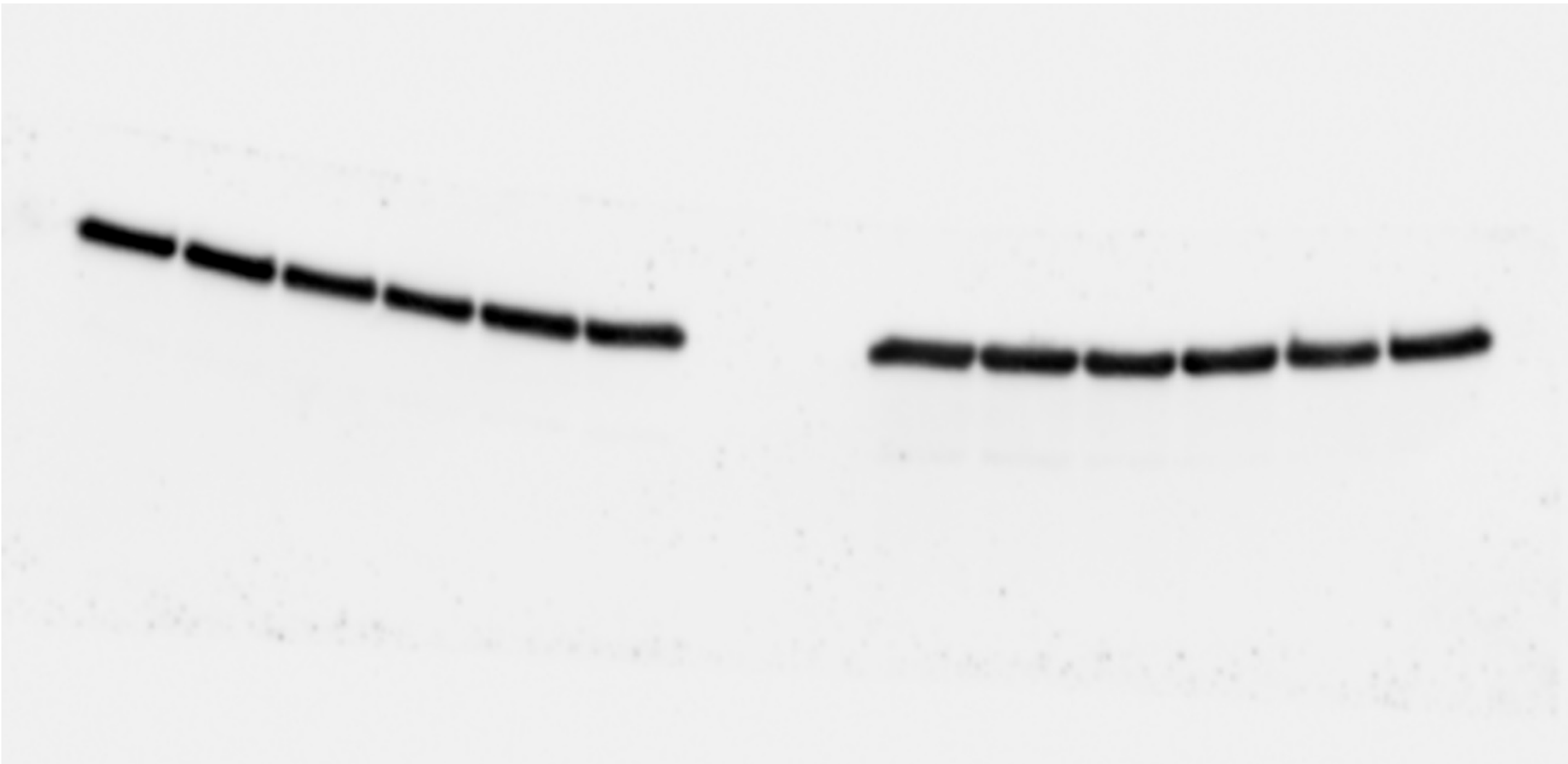

GAPDH/MR42D GAPDH/PC3
